# Enantioselective Synthesis of a New Non-Natural Gabosine

**DOI:** 10.3390/molecules26051423

**Published:** 2021-03-06

**Authors:** Maximiliano Colobbio, Enrique Pandolfi, Valeria Schapiro

**Affiliations:** Laboratorio de Síntesis Orgánica, Departamento de Química Orgánica, Facultad de Química, Universidad de la República, General Flores 2124, Montevideo CP 11800, Uruguay; mcolobbio@gmail.com (M.C.); epandolf@fq.edu.uy (E.P.)

**Keywords:** gabosine, chemoenzymatic, enantioselective synthesis

## Abstract

The preparation of a new non-natural gabosine is reported, in which the chirality is transferred from the toluene’s biotransformed metabolite (1*R*,2*S*)-*3*-methylcyclohexa-3.5-diene-1,2-diol. Further chemical transformations to introduce additional functionality and chirality to the molecule were also accomplished.

## 1. Introduction

Monosubstituted benzenes can be enzymatically transformed into chiral diols [1,2,3] that are valuable synthons for enantioselective synthesis. They have been widely and successfully used for the preparation of natural products and analogs with biological properties of interest [4,5].

**Figure 1:**
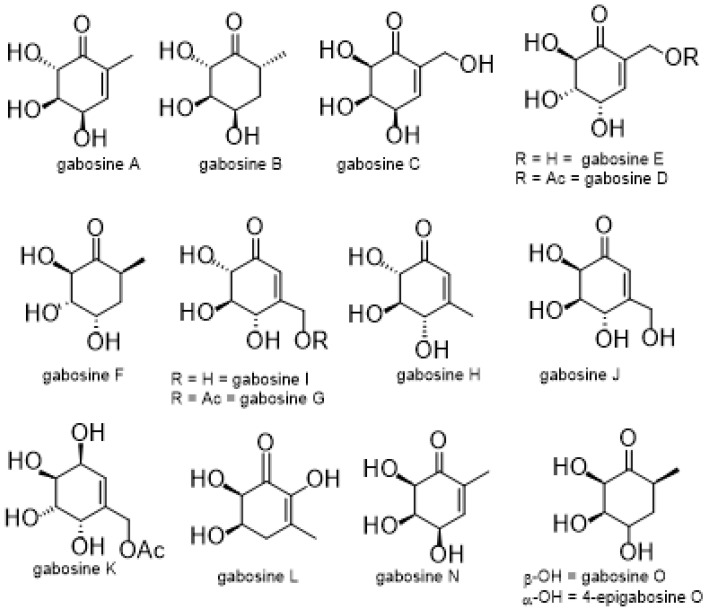
The gabosine family.

In this field, we have focused on gabosines and anhydrogabosines. Gabosines (Figure 1) are keto-carbasugars bearing a methyl or hydroxymethyl side chain, originally described in 1974 [6,7]. They show promising pharmacological activities as antibiotic, anticancer, enzyme inhibition, and DNA-binding properties [8,9,10,11,12,13,14].

**Figure 2:**
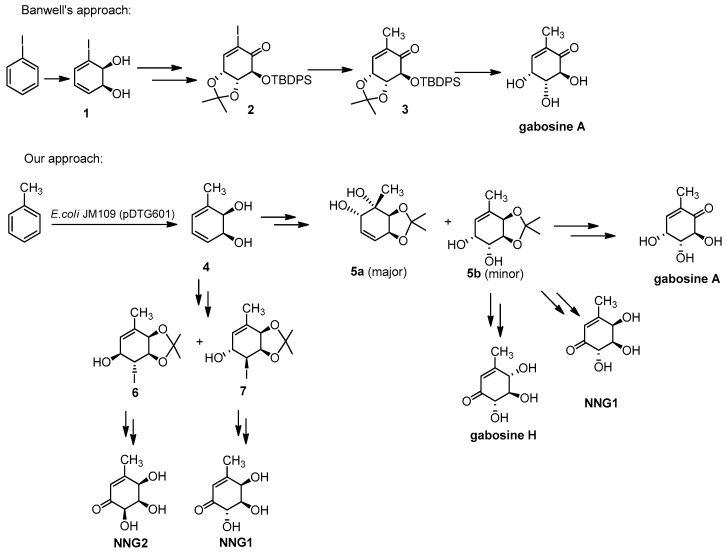
Gabosine’s syntheses from biotransformation of monosubstituted benzenes.

This wide range of biological properties turned the gabosine family into a group of coveted synthetic targets. During the last 30 years, several strategies have been developed to prepare gabosines [15,16,17,18,19,20,21,22,23,24,25,26,27,28,29,30] and related derivatives [31,32,33,34,35,36,37,38]. Among these and other synthetic efforts, only Banwell’s group and ours have applied biotransformations to introduce chirality into the process. Although toluene is the most suitable aromatic substrate, as it bears the methyl side chain of some gabosines, in 2001, Banwell and co-workers failed in their attempt to use it, and reported the preparation of gabosine A from iodobenzene derived diol **1** [39], introducing the methyl group in one of the lasts steps, as shown in Figure 2. In 2011, we were able to prepare the same target from toluene [40] and in 2017 we reported the synthesis of gabosine H and two non-natural methylgabosines [41] from the same starting material (Figure 2).

A key intermediate in our syntheses of gabosines A and H was compound **5b**. This compound is the minor product obtained during osmilation of toluene diol acetonide, which is a technique extensively used in our lab to functionalize the diene. Toluene derived diols reacts preferentially on the more substituted olefine, leading to **5a** [42] and consequently **5b**, the usually unwanted minor regioisomer, is readily available to us. We have already reported the synthesis of a non-natural gabosine (**NNG1**) from compound **5b** [41]. 

**Figure 3:**
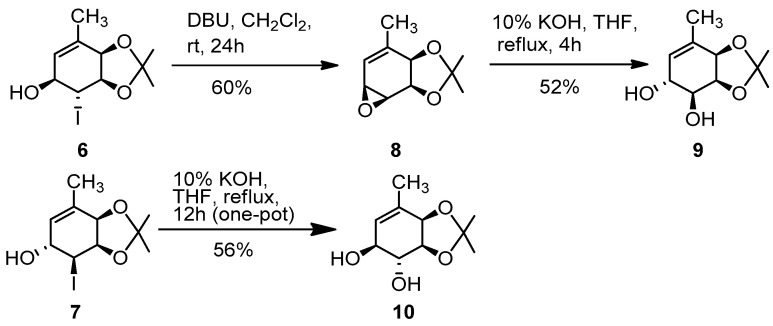
Reported conversion of iodohydrins into diols.

To prepare **NNG2**, along with an alternative route for **NNG1**, previously, we took advantage of another methodology extensively studied and used in our lab: the hydroxyhalogenation of the cyclohexadiendiols [43]. Using both the major (**6**) and the minor (**7**) products, we reported the preparation of **NNG1** and **NNG2**. Major iodohydrin **6** was converted into **NNG2** and minor iodohydrin **7** was converted into **NNG1**. In both cases, the first step was the substitution of the iodine atom by a hydroxy group, through a sequence of epoxide formation and opening [44]. In that previous report, we essayed a stepwise sequence, as well as a one-pot reaction, as shown in Figure 3.

**Figure 4:**
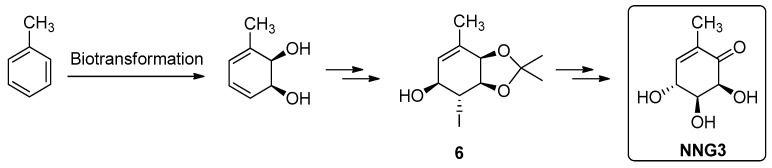
Proposed strategy for the synthesis of **NNG3.**

Herein, we focused on the challenge of synthesizing a new non-natural gabosine, **NNG3**, an epimer of gabosine A, as shown in Figure 4. 

The preparation of a new gabosine, **NNG3,** confirms the efficiency and usefulness of our methodology, using toluene as starting material.

## 2. Results

First, we decided to broad and optimize the use of the hydroxyhalogenation to prepare non-natural gabosines, starting from major iodohydrin **6** (prepared from diol **4** in 2 steps, 77% overall yield) [43]. The first goal was to improve the yield of our own previous work: the transformation of iodohydrins **6** and **7** into diols **9** and **10** respectively, as shown in Figure 3 (31% for **9** in two steps from **6**, 56% for **10** in one step from **7**) [41]. Table 1 displays the results achieved for the conversion of **6** into **9** in one step performed in a 3:1 THF:H_2_O mixture.

During these experiments, we observed that diol **9** was not stable under chromatographic purification conditions. Therefore, we first assayed the protection of purified **9**, and then a “tandem” procedure was also explored. At this stage, we were pleased to verify an increase of the yield from 64% to 90% taking advantage of the “tandem” strategy.

**Figure 5:**
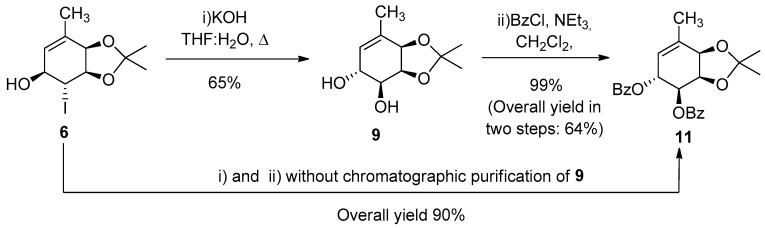
Optimization of key intermediate **11**’s preparation.

These results are summarized in Figure 5.

**Figure 6:**
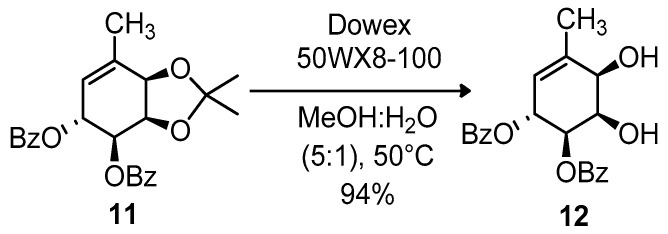
Selective deprotection of **11**.

Next, the acetonide group was selectively removed from compound **11** by means of an acidic resin (Figure 6).

**Figure 7:**
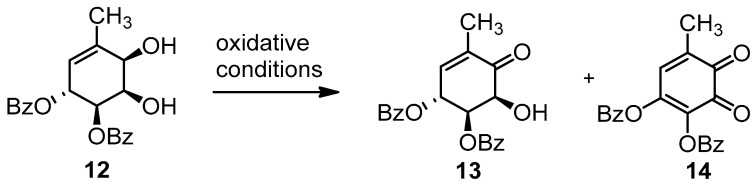
Selective oxidation of diol **12**.

In the following step, the oxidation of the allylic alcohol, was unexpectedly laborious, mainly due to the formation of an over-oxidized product (Figure 7).

We assayed a series of oxidizing agents, with poor-to-moderate results, which are summarized in Table 2.

Initially, we used IBX, a reagent routinely employed by our group for these transformations [40] (Table 2, entry 1). However, we were surprised to observe the formation of the diene-dione **14** along with the target product **13**. This fact led us to switch the oxidizing agent to PCC with acceptable results (Table 2, entry 2). We attempted to improve this yield, based on preventing the adsorption of the products on SiO_2_. We decreased the equivalents of PCC, diluted the reaction mixture and replaced SiO_2_ with molecular sieves (Table 2, entries 3 and 4) [19] with poor results. Attempts to improve on these results using SO_3_.Py [45] (Table 2, entry 5), MnO_2_ (Table 2, entries 6 and 7) [46], NBS and IBX in the presence of cyclodextrin [47,48](Table 2, entries 8 and 9) and in situ generation of IBX with Oxone [49] (Table 2, entries 10 and 11) led to lower yields. Consequently, we reverted to the use of IBX and PCC. Since IBX is a safer reagent and the formation of by-products is minimized compared to PCC, we decided to focus on this oxidizing agent. Following methodology on the influence of solvents in oxidations using IBX [50], we improved the yield in the preparation of **13**. These results are shown in Table 3.

**Figure 8:**
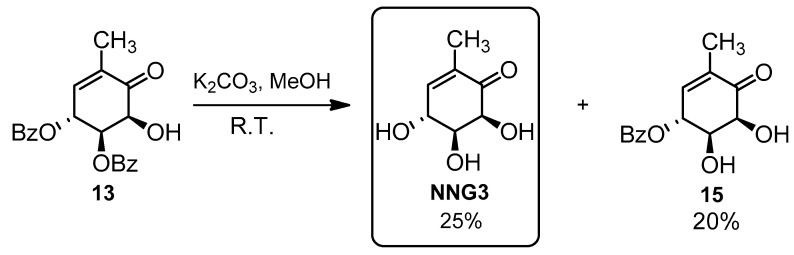
Basic deprotection of **13**.

Although the use of polar solvents in which IBX is more soluble (Table 3, entries 1–4) allowed working at lower temperatures, this did not avoid the formation of over-oxidized compound **14**, thereby not increasing the yield of **13** significantly. The use of AcOEt was not useful either (Table 3, entries 5–7). The best results were achieved in acetone (Table 3, entries 8–13) with strict chromatographic monitoring of unwanted **14** to stop the reaction (Table 3, entry 8). These efforts permitted to enhance yields and reduce reaction times, as well as to prevent the formation of decomposition products and thus facilitate the further purification steps. The final step was the benzoyl groups removal. We were aware of previous reports concerning the instability of gabosines in basic medium, including the first gabosine’s isolation [6]. In Banwell´s chemoenzymatic approach [39], the authors describe gabosines as “base-sensitive compounds”. Despite this, we first decided to perform the classic basic methanolysis, noticing evident decomposition of the starting material **13**, low yield for the final product **NNG3** and partial deprotection (Figure 8).

**Figure 9:**
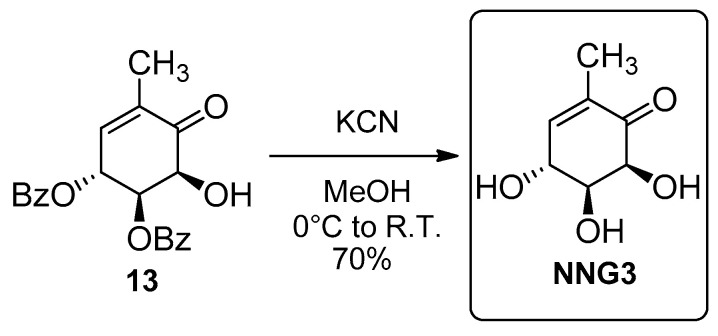
Synthesis of **NNG3**.

However, none of the attempts to deprotect the diol in acid medium succeeded. Most of them (5% HCl in methanol, catalytic BF_3_.Me_2_S in CH_2_Cl_2_ and Dowex 50W8 strongly acid in HCl 1M) led to 100% decomposition, and when treated with trifluoroacetic acid in the presence of water, we only recovered the starting material. Deprotection was finally successful by means of KCN [52], as shown in Figure 9.

The experiments concerning the structural elucidation of all new compounds can be consulted in the Supplementary Materials section.

## 3. Materials and Methods

### 3.1. Chemicals and Materials

Commercially available reagents were purchased and used without further purification. Solvents were distilled prior to use. Non-hydrolytic reactions were carried out in nitrogen atmosphere with standard techniques for the exclusion of air. Reactions were monitored by thin-layer chromatography (TLC) performed on plates pre-coated with silica gel 60F 254 and visualized with UV light (254 nm) and/or *p*-anisaldehyde in acidic ethanolic solution and/or vanillin in acidic ethanolic solution. Flash column chromatography was performed using silica gel (Kieselgel 60, EM reagent, 230–400 mesh (Macherey-Nagel, Düren, Germany) The ratio of the eluents is informed in volume in all cases. Yields refer to chromatographically and spectroscopically homogeneous materials. 

### 3.2. Instrumentation

Melting points were recorded in Fisher-Johns melting point apparatus serial 4446 Fisher-Scientific company (Fisher-Scientific international Inc., Pittsbugh, PA, USA) and are uncorrected. Mass spectra were recorded, using the electron impact mode (70 or 20 eV) in a GCMS QP2010 Shimadzu HP 5971 spectrometer (Shimadzu Corporation, Kyoto, Japan). Elementary analyses were performed in a microanalyzer Fisons EA 1108 CHNS-O (Fison Instruments Ltd., Glasglow, UK) Infrared spectra were recorded on neat samples (NaCl disks), using a Shimadzu FT/IR-8101 Type A spectrometer (Shimadzu Corporation, Kyoto, Japan). NMR spectra were obtained in a Bruker Advance DPX-400 instrument at 400 MHz for ^1^H NMR and 100 MHz for ^13^C NMR in CDCl_3_ or MeOH-D_4_ or (CD_3_)_2_CO (Bruker Corporation, Billerica, MA, USA). Proton chemical shifts are reported in ppm downfield from TMS as an internal reference, and carbon chemical shifts are reported in ppm relative to the center line of the CDCl_3_ triplet (77.0 ppm). Optical rotations were measured in a Krüss Optronic P8000 polarimeter (A. Krüss Optronic GmbH, Hamburg, Germany), using a 0.5 dm cell. Moreover, [α]_D_ values are given in units of deg cm^2^ g^−1^, and concentration values are expressed in g/100 mL.

### 3.3. Experimental Procedures

#### 3.3.1. Synthesis of (1R,2R,5R,6S)-1,2-O-isopropyliden-3-methylciclohex-3-en-1,2,5,6-tetraol (9) 

Compound **6** (1.3 g, 3.9 mmol) was dissolved in a THF:H_2_O mixture (3:1, 29 mL), and KOH (1.8 g, 32 mmol) was added. The mixture was heated under reflux for 8 h, cooled to room temperature, and poured on H_2_O (80 mL). The aqueous phase was extracted with AcOEt (1 × 50 mL, 4 × 25 mL), and the combined organic layers were first washed with saturated NH_4_Cl (1 × 25 mL) and then with saturated NaCl (1 × 25 mL). After drying over Na_2_SO_4_ and evaporating the solvent under vacuum, the product was purified by flash column chromatography, in SiO_2_, using 7:3 AcOEt:hexanes, to afford **9** (508 mg, 65%) as a white solid. All spectroscopic and physical properties are in concordance with the reported data.

#### 3.3.2. Synthesis of (1R,2R,5R,6S)-5,6-dibenzoyl-1,2-O-isopropyliden-3-methylciclohex-3- en-1,2,5,6-tetraol (11)

(a) One step from **9**:

Compound **9** (508 mg, 2.58 mmol) was dissolved in CH_2_Cl_2_ (25 mL), under nitrogen, and cooled 0 °C. NEt_3_ (1.4 mL, 10.3 mmol) was added and after a few minutes, and then BzCl (0.75 mL, 6.45 mmol) was slowly dropped into the mixture. The system was allowed to reach room temperature and stirred for 24 h; the mixture was then poured on saturated NaHCO_3_ (50 mL) and extracted with CH_2_Cl_2_ (3 × 30 mL). The combined organic layers were first washed with saturated NH_4_Cl (1 × 20 mL) and then with saturated NaCl (1 × 20 mL). After drying over Na_2_SO_4_ and evaporating the solvent under vacuum, the product was purified by flash column chromatography, in SiO_2_, using 4:6 CH_2_Cl_2_:hexanes, to afford **11** (1.04 g, 99%) as a white foamy solid.

(b) “Tandem” synthesis from **6**:

The procedures described in Section 3.3.1 and Section 3.3.2. (a) are followed one after the other, with no chromatographic purification of **9**, starting from **6** (600 mg, 1.94 mmol). **11** obtained as a foamy white solid (788 mg, 90% overall)

^1^H-NMR (400 MHz, CDCl_3_) δ(ppm): 8.07–7.97 (m, 4H), 7.50–7.41 (*m*, 2H), 7.40–7.36 (*m*, 4H), 6.12–6.08 (*m*, 1H), 5.55–5.54 (*m*, 2H), 4.69 (*dd*, *J_1_* = 5.0, *J_2_* = 2.5 Hz, 1H), 4.59 (*dd*, *J_1_* = 5.1, *J_2_* = 1.8 Hz, 1H), 1.86 (s, 3H), 1.44 (*s*, 3H), 1.38 (*s*, 3H); ^13^C- NMR(101 MHz, CDCl_3_) δ(ppm):166.27,166.10, 136.49, 133.27, 133.11, 130.00, 129.85, 129.70, 129.56, 128.38, 121.61, 110.65, 76.83, 74.36, 72.45, 69.70, 27.59, 26.82, 19.12; EA: 70.6189% C (calculated 70.5882%, |δ= 0.0307%), 5.7118% H (calculated 5.8824%, |δ= 0.1706%); M.P. = (50–52) °C; [α]_D_^19^ = −183.1 (c 0.855, acetone); IR (NaCl)/cm^−1^: 3062, 2985, 1712, 1602, 1585, 1490, 1315, 1236, 1178,1161, 1111, 995, 886.

#### 3.3.3. Synthesis of (1R,2R,5R,6R)-5,6-dibenzoyl-3-methylciclohex-3-en-1,2,5,6-tetraol (12)

Compound **11** (277 mg, 0.68 mmol) was dissolved in a mixture of MeOH:H_2_O (5:1, 4.3 mL), acid resin Dowex 50wx8-100 (1.1 g) was added, and the system was stirred at 50 °C for 24 h. Once all the starting material was consumed, the resin was vacuum filtered and washed several times with methanol, until the product was no longer detectable in the filtrate by TLC. Methanol was evaporated under vacuum and water was also eliminated by vacuum distillation in the presence of a few drops of AcOEt (azeotropic distillation). The product was purified by flash column chromatography in SiO_2_, using 7:3 AcOEt:hexanes, to afford **12** (233 mg, 94%) as a foamy white solid.

^1^H-NMR (400 MHz, CDCl_3_) δ(ppm): 8.02–7.97 (*m*, 4H), 7.52–7.50 (*m*, 2H), 7.40–7.37 (*m*, 4H), 6.08–6.05 (*m*, 1H), 5.60 (*t*, *J* = 1.2 Hz, 1H), 5.46 (*dd*, *J_1_* = 7.5, *J_2_* = 2.0 Hz, 1H), 4.46 (*dd*, *J_1_* = 3.9, *J_2_* = 2.0 Hz, 1H), 4.34 (*d*, *J* = 3.7 Hz, 1H), 3.14 (*s*, 1H), 2.74 (*s*, 1H), 1.89 (*s*, 3H); ^13^C-NMR (101 MHz, CDCl_3_) δ(ppm): 166.17, 166.05, 138.53, 133.44, 133.21, 129.81, 129.73, 129.36, 128.52, 128.40, 120.71, 74.28, 70.45, 70.18, 69.99, 19.32; EA: 68.2454% C (calculated 68.4783%, |δ = 0.2329%), 5.6997% H (calculated 5.4348%, |δ = 0.2649%); M.P. = (70–72) °C; [α]_D_^19^ = −222.6 (c 0.540, acetone); IR (NaCl)/cm^−1^:3396, 3091, 3070, 2953, 2976, 2918, 1693, 1645, 1633, 1602, 1585, 1452, 1276, 1178, 1111, 1026, 985, 706.

#### 3.3.4. Synthesis of (4R,5R,6S)-4,5-dibenzoyl-6-hydroxy-2-methylciclohex-2-enone (13)

(a) Procedure using PCC/SiO_2_:

In a mortar, PCC (60 mg) and SiO_2_ (60 mg) were grinded until a homogeneous fine orange mixture was obtained. This solid was placed in a round-bottom flask, CH_2_Cl_2_ (28 mL) was added, and the mixture was stirred for a few minutes. Then **12** (40 mg) was added, the solution got darker, and after 45 min, the reaction was stopped. Et_2_O (15 mL) was added, and then it was vacuum filtered through a double celite and SiO_2_ layer. The filter aid was washed with Et_2_O, until the product was no longer detectable in the filtrate by TLC. The solvent was removed by vacuum distillation, and the crude product was re-dissolved in Et_2_O (15 mL). The organic phase was washed with NaHCO_3_ (1 × 5 mL) y NaCl (1 × 5 mL) and then dried over Na_2_SO_4_. The solvent was vacuum distilled, and the product was purified by flash column chromatography, in SiO_2_, using 1:9 AcOEt:hexanes, to afford **13** (20.4 mg, 51%) as a yellowish solid.

(b) Procedure using SO_3_Py:

Compound **12** (64 mg, 0.17 mmol) was dissolved in a mixture of CH_2_Cl_2_:DMSO (3:1, 1.3 mL), under nitrogen. The mixture was cooled to 0 °C, and then NEt_3_ (0.15 mL, 1.054 mol) and the complex SO_3_Py (145 mg, 0.561 mmol) were added. The mixture was allowed to reach room temperature, until no more starting material was detected (3 h), and then it was diluted with CH_2_Cl_2_ (20 mL) and washed with H_2_O (20 mL). The aqueous layer was extracted with CH_2_Cl_2_ (3 × 10 mL). The combined organic layers were dried over Na_2_SO_4_. The solvent was vacuum distilled and the product was purified by flash column chromatography in SiO_2_ using 1:9 AcOEt:hexanes to afford **13** (23 mg, 36%) as a yellowish solid.

(c) Procedure using MnO_2:_

Compound **12** (50.0 mg, 0.13 mmol) was dissolved in CH_2_Cl_2_ (3.0 mL) and cooled to −20 °C. MnO_2_* (700 mg, 8.06 mmol) was added in portions and the mixture was stirred for 24 h. The reaction mixture was vacuum filtered through celite, washing with CH_2_Cl_2_. The solvent was vacuum distilled, and the product was purified by flash column chromatography in SiO_2_, using 1:9 AcOEt:hexanes, to afford **13** (12 mg, 29%) as a yellowish solid.

Preparation of MnO_2_: KMnO_4_ (4.7 g) was dissolved in H_2_O (30 mL), and a solution of MnSO_4_^.^H_2_O (5 g) in H_2_O (30 mL) was slowly added, drop by drop. After an hour, the precipitate was filtered; washed with H_2_O, at 0 °C; and dried in an oven, at 120 °C, for 24 h (81%). 

(d) Procedure using 2-IBA-Oxone

Compound **12** (89.0 mg, 0.24 mmol) was dissolved in a mixture MeCN:H_2_O (2:1, 3.1 mL). The, 2-Iodobenzoic acid (36.0 mg, 0.072 mmol) and Oxone (738 mg, 1.2 mmol) were added. The mixture was heated to 70 °C and stirred for 16 h; it was then cooled to 0 °C and filtered. The precipitate was washed twice with H_2_O and with CH_2_Cl_2_, until the product was no longer detectable in the filtrate by TLC. The aqueous phase was extracted with CH_2_Cl_2_ (3 × 5mL)_._ The combined organic layers were dried over Na_2_SO_4_. The solvent was vacuum distilled, and the product was purified by flash column chromatography in SiO_2_, using a gradient of AcOEt:hexanes (from 1:99 to 2:8), to afford **13** (30 mg, 34%) as a yellowish solid.

(e) Optimized procedure using IBX

Compound **12** (59.4 mg, 0.16 mmol) was dissolved in acetone (5.4 mL), IBX* (136 mg, 0.49 mmol) was added, and the mixture was heated under reflux. The appearance of a yellow-to-orange color in 45 min indicates the formation of over-oxidized product **14**. Thus, after 45 min, the reaction was allowed to cool to room temperature, and AcOEt (10 mL) was added. The mixture was filtered, and the solid was washed with AcOEt, until the product was no longer detectable in the filtrate by TLC. The organic phase was washed with saturated NaHSO_3_ (1 × 10 mL) and saturated NaCl (1 × 5 mL). The combined organic layers were dried over Na_2_SO_4_. The solvent was vacuum distilled, and the product was purified by flash column chromatography, in SiO_2_, using a gradient of AcOEt:hexanes (from 1:99 to 2:8), to afford **13** (33 mg, 56%) as a yellowish solid.

^1^H-NMR (400 MHz, CDCl_3_) δ(ppm): 8.08–8.06 (*m*, 2H), 7.92–7.90 (*m*, 2H), 7.62–7.55 (*m*, 2H), 7.46 (*t*, *J* = 7.7 Hz, 2H), 7.44 (*t*, *J =* 7.7 Hz, 2H), 6.79 (*dq*, *J_1_* = 5.0, *J_2_* = *J_3_ = J_4_* = 1.6 Hz, 1H), 5.91–5.90 (*m*, 1H), 5.80–5.79 (*m*, 1H), 4.98 (*t*, *J* = 2.9 Hz, 1H), 3.57 (*d*, *J* = 3.2 Hz, 1H), 2.01 (*t*, *J* = 1.3 Hz, 3H); ^13^C- NMR (101 MHz, CDCl_3_) δ(ppm):197.47, 165.07, 137.75, 135.96, 133.75, 133.57, 129.92, 129.86, 129.07, 128.98, 128.63, 128.50, 73.36, 71.25, 67.11, 15.61; E.A.: 68.7589% C (calculated 68.8852%, |δ = 0.1263%), 5.2039%H (calculated 4.9180%, |δ = 0.2859%): M.P. = (120–124) °C (decomposes); [α]_D_^19^ = −168.6 (c 0.535, acetone); IR (NaCl)/cm^−1^:3446, 3093, 3070, 2959, 1720, 1697, 1647, 1601, 1585, 1491, 1452, 1315, 1261, 1178, 1095, 1070, 710.

Preparation of IBX: [51] 2-iodobenzoic acid (2.5 g, 0.050 mol) was added to a solution of Oxone in distilled water (18.6 g, 0.031 mol, 3 eq/0.2 L of H_2_O). The suspension was heated to 70 °C and allowed to react until turbidity disappeared, or for 3 h. Then, the mixture was cooled to 0–5 °C, and the precipitate was vacuum filtered. The white solid was washed with H_2_O (6 × 5 mL) and then with acetone (2 × 5 mL); allowed to dry for 2 h, at room temperature; and stored at −20 °C, protected from light and under nitrogen (1.78 g, 64%).

#### 3.3.5. Synthesis of (4R,5S,6S)-4,5,6-trihidroxy-2-methylcyclohex-2-enone (NNG3)

(a) Procedure using K_2_CO_3_

Compound **13** (45 mg, 0.12 mmol) was dissolved in dry MeOH (4.7 mL), under nitrogen, cooled to 0 °C, and a catalytic amount of K_2_CO_3_ (2–4 mg), previously dried in an oven, at 120 °C, was added. The mixture was allowed to reach room temperature and stirred for 5 days. It progressively darkened until it turned black, thus indicating the decomposition of the product. The crude mixture was filtered through dry SiO_2_, which was washed with AcOEt:MeOH (7:3), until the product was no longer detectable in the filtrate by TLC. The solvent was vacuum distilled, and the product was purified by flash column chromatography, in SiO_2_, using a gradient of AcOEt:hexanes (from 8:2 to AcOEt 100%), to afford NNG3 (5 mg, 25%) as white solid.

(b) Procedure using KCN

Compound **13** (50.4 mg, 0.14 mmol) was dissolved in dry MeOH (1.4 mL), under nitrogen, cooled to 0 °C, and then KCN (18.2 mg, 0.28 mmol) was added. The mixture was allowed to reach room temperature and stirred for 4 h. The appearance of a reddish color was observed. Then, Ion exchange resin “Ion exchanger IV” (36.0 mg) was added to the reaction mixture and stirred for several minutes. The resin was filtered and washed with MeOH (3 × 5 mL). The crude mixture was filtered through dry SiO_2_, which was washed with AcOEt:MeOH (7:3), until the product was no longer detectable in the filtrate by TLC. The solvent was vacuum distilled, and the product was purified by flash column chromatography, in SiO_2_, using AcOEt to afford **NNG3** (15.1 mg, 70%) as a white solid. 

^1^H-NMR (400 MHz, (C_3_D_3_)_2_O) δ(ppm):6.44 (*dt*, *J_1_* = 4.7, *J_2_* = 1.6 Hz, 1H), 4.44 (*d*, *J* = 5.5 Hz, 1H), 4.39–4,37 (*m*, 1H), 4.26–4.22 (*md*, 1H), 4.12 (*d*, *J* = 3.7 Hz, 1H), 4.01–3.99 (*m*, 1H), 3.91 (*d*, *J* = 4.0 Hz, 1H), 1.64 (*t*, *J* = 1.4 Hz, 3H); ^13^C-NMR(101 MHz, (C_3_D_3_)_2_O) δ(ppm): 199.01, 141.11, 133.67, 75.24, 73.02, 68.13, 14.49; EA: 51.2868% C (calculated 53.1617%, |δ = 1.8749%), 6.6664% H (calculated 6.3727%, |δ = 0.2937%) M.P. = (118–120) °C; [α]_D_^20^ = −34.5 (c 0.18, acetone); IR (NaCl)/cm^−1^: 3431, 2955, 2922, 2851, 1640, 1632.

## 4. Conclusions

We succeeded in preparing a new non-natural gabosine, with potential bioactivity, in an enantiomerically pure form. This was possible starting from a biotransformation of toluene that introduces chirality to the process, in seven steps, from diol 4, with 25% overall yield. Exhaustive methodological work was conducted, especially for the optimization of the selective oxidation step, along with the iodine-substitution reaction and the final deprotection. Further application of this methodology in the synthesis of new gabosines and analogs, bioactivity assays, and crystallographic analyses will be reported in due course.

## Figures and Tables

**Table 1:** Conditions assayed for the preparation of diol **9** in one step.

Entry	Base	Equivalents	[6] (M)	T (°C)	Time (h)	Products (Yield) *
1	NaOH	4.0	0.18	R.T.-reflux	24 h R.T.+ 4 h reflux	**9** (55%) + **8** (10%)
2	NaOH	4.0	0.20	reflux	6	**9** (42%)
3	NaOH	4.0	0.15	reflux	48	**9** (54%)
4	NaOH	5.0	0.18	reflux	6	**9** (45%)
5	NaOH	6.0	0.15	reflux	8	**9** (58%) + **8** (8%)
6	KOH	4.0	0.15	reflux	9	**9** (61%)
7	KOH	6.6	0.15	reflux	10	**9** (55%)
8	KOH	8.0	0.15	reflux	8	**9** (65%)

R.T: room temperature * The yields correspond to chromatographically purified products.

**Table 2:** Conditions for the selective oxidation of **12**.

Entry	Oxidizing Agent (Equivalents)	Solvent	[12] (M)	T (°C)	Time (h)	Products (Yield) *
1	IBX (2.0)	DMF	0.10	0–R.T.	48	**13** (45%) + **14**
2	PCC/SiO_2_ (2.0)	CH_2_Cl_2_	0.15	R.T.	0.75	**13** (51%)
3	PCC/SiO_2_ (1.5)	CH_2_Cl_2_	0.05	R.T.	0.75	**13** (26%)
4	PCC/molecular sieves 4Å (2.0)	CH_2_Cl_2_	0.22	R.T.	1.5	**14** + D.P.
5	SO_3_^.^Py (3.1), NEt_3_ (6.2)	CH_2_Cl_2_:DMSO (3:1)	0.15	0–R.T.	3	**13** (36%) +**14**
6	MnO_2_ (10% m-m)	CHCl_2_	0,05	0–R.T.	0.25	**14** + D.P.
7	MnO_2_ (14% m-m)	CHCl_2_	0.05	−20	24	**13** (29%) + **14**
8	NBS (3.0), β-cyclodextrin (1.0)	H_2_O	0.07	R.T.	48	**14** + D.P.
9	IBX (1.1), β-cyclodextrin (0.1)	H_2_O:acetone (86:14)	0.07	R.T.	24	**12**
10	2-IBA (0.6), Oxone (5.0)	MeCN:H_2_O (2:1)	0.08	R.T.	96	**12**
11	2-IBA (0.6), Oxone (5.0)	MeCN:H_2_O (2:1)	0.08	70	16	**13** (34%)

* The yields correspond to chromatographically purified products. The yields of secondary product **14** could not be determined. D.P. = decomposition products.

**Table 3:** Conditions for the selective oxidation of **12**, using IBX prepared according to Frigerio et al. [51].

Entry	IBX (eq.)	Solvent	[12] (M)	T (°C)	Time (h)	Products (Yield) *
1	2.0	DMF	0.10	0–R.T.	48	**13**(45%) + **14**
2	3.0	DMF	0.10	0–R.T.	24	**13**(22%) + **14**
3	3.0	DMF	0.25	0–R.T.	1.0	**14** + D.P
4	1.5	DMSO	0.35	0–R.T.	0.33	**14** + D.P.
5	3.0	AcOEt	0.10	40	23	**13**(10%) + **14**
6	3.0	AcOEt	0.03	R.T.	72	**14** + D.P.
7	3.0	AcOEt	0.03	reflux	4	**14** + D.P.
8	3.0	Acetone	0.02	reflux	2	**13**(52%) + **12**
9	3.0	Acetone	0.03	reflux	0.75	**13**(56%) + **12**
10	3.0	Acetone	0.03	reflux	1.5	**13**(36%) + **14**
11	3.0	Acetone	0.03	R.T.	48	**14** + D.P.
12	2.0	Acetone	0.03	reflux	0.83	**13**(38%) + **14**
13	1.5	Acetone	0.03	reflux	24	**13**(24%) + **12**

eq.: equivalents * The yields correspond to chromatographically purified products. The yields of secondary product **14** could not be determined. D.P. = decomposition products.

## Data Availability

The data presented in this study are available within the article and in the Supplementary Materials section.

## Supplementary Materials

Figure S1: ^1^H-NMR spectrum of **11**. Figure S2: Extended ^1^H-NMR spectrum of **11 (I)**. Figure S3: Extended ^1^H-NMR spectrum of **11 (II)**. Figure S4: Extended ^1^H-NMR spectrum of **11 (III)**. Figure S5: Extended ^1^H-NMR spectrum of **11 (IV)**. Figure S6: Extended ^1^H-NMR spectrum of **11 (V)**. Figure S7: Extended ^1^H-NMR spectrum of **11 (VI)**. Figure S8: ^13^C-NMR spectrum of **11**. Figure S9: IR spectrum of **11**. Figure S10: ^1^H-NMR spectrum of **12**. Figure S11: Extended ^1^H-NMR spectrum of **12 (I)**. Figure S12: Extended ^1^H-NMR spectrum of **12 (II)**. Figure S13: Extended ^1^H-NMR spectrum of **12 (III)**. Figure S14: Extended ^1^H-NMR spectrum of **12 (IV)**. Figure S15: Extended ^1^H-NMR spectrum of **12 (V)**. Figure S16: Extended ^1^H-NMR spectrum of **12 (VI)**. Figure S17: ^13^C-NMR spectrum of **12**. Figure S18: IR spectrum of **12**. Figure S19: ^1^H-NMR spectrum of **13**. Figure S20: Extended ^1^H-NMR spectrum of **13 (I)**, Figure S21: Extended ^1^H-NMR spectrum of **13 (II).** Figure S22: Extended ^1^H-NMR spectrum of **13 (III)**. Figure S23: Extended ^1^H-NMR spectrum of **13 (IV).** Figure S24: Extended ^1^H-NMR spectrum of **13 (V),** Figure S25: Extended ^1^H-NMR spectrum of **13 (VI).** Figure S26: ^13^C-NMR of **13**. Figure S27: IR spectrum of **13**. Figure S28 ^1^H-NMR spectrum of **14**. Figure S29: COSY experiment of **14**. Figure S30: ^1^H-NMR spectrum of **NNG3**. Figure S31: Extended ^1^H-NMR spectrum of **NNG3 (I).** Figure S32: Extended ^1^H-NMR spectrum of **NNG3 (II).** Figure S33: Extended ^1^H-NMR spectrum of **NNG3 (III)**. Figure S34: Extended ^1^H-NMR spectrum of **NNG3 (IV)**. Figure S35: COSY experiment of **NNG3**. Figure S36: ^13^C-NMR spectrum of **NNG3**. Figure S37: HSQC experiment of **NNG3**. Figure S38: HMBC experiment of **NNG3**. Figure S39: IR spectrum of **NNG3**. Figure S40: ^1^H-NMR spectrum of **15**. Figure S41: COSY experiment of **15**.

## Sample Availability

Samples of the compounds are available from the authors.

## Abbreviations

NNG	Non-natural gabosine
TBDPS	Tert-butyl-diphenylsilane
DBU	1,8-Diazabicycle [5.4.0]-undec-7ene
THF	Tetrahydrofuran
IBX	Iodoxybenzoic acid
PCC	Pyridinium chlorochromate
DMF	Dimethylformamide
DMSO	Dimethylsulfoxide
IBA	Iodobenzoic acid
