# Supplementary material for: Enantioselective Synthesis of a New Non-Natural Gabosine
Source: Molecules. 2021 Mar 6;26(5):1423. doi: 10.3390/molecules26051423 (PMC7961443; doi:10.3390/molecules26051423)

SUPPLEMENTARY MATERIALS

Article

Enantioselective Synthesis of a New non-Natural Gabosine

Maximiliano Colobbio, Enrique Pandolfi and Valeria Schapiro *

Laboratorio de Síntesis Orgánica, Departamento de Química Orgánica, Facultad de Química, Universidad de la República, General Flores 2124, Montevideo, CP 11800, Uruguay; mcolobbio@gmail.com, epandolf@fq.edu.uy

* Correspondence: vschapir@fq.edu.uy; Tel.: +598-2-9247881

1) *(1R,2R,5R,6S)-5,6-dibenzoyl-1,2-O-isopropyliden-3-methylciclohex-3- en-1,2,5,6-tetraol* (**11**)

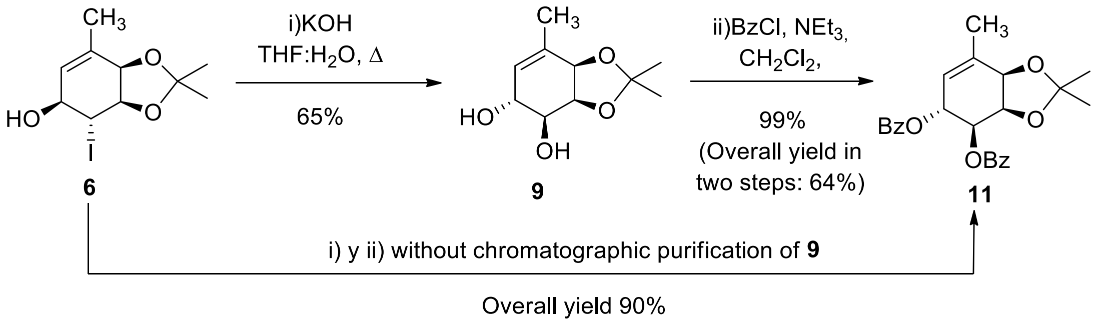

Figure S1. ^{1}H-NMR spectrum of **11**

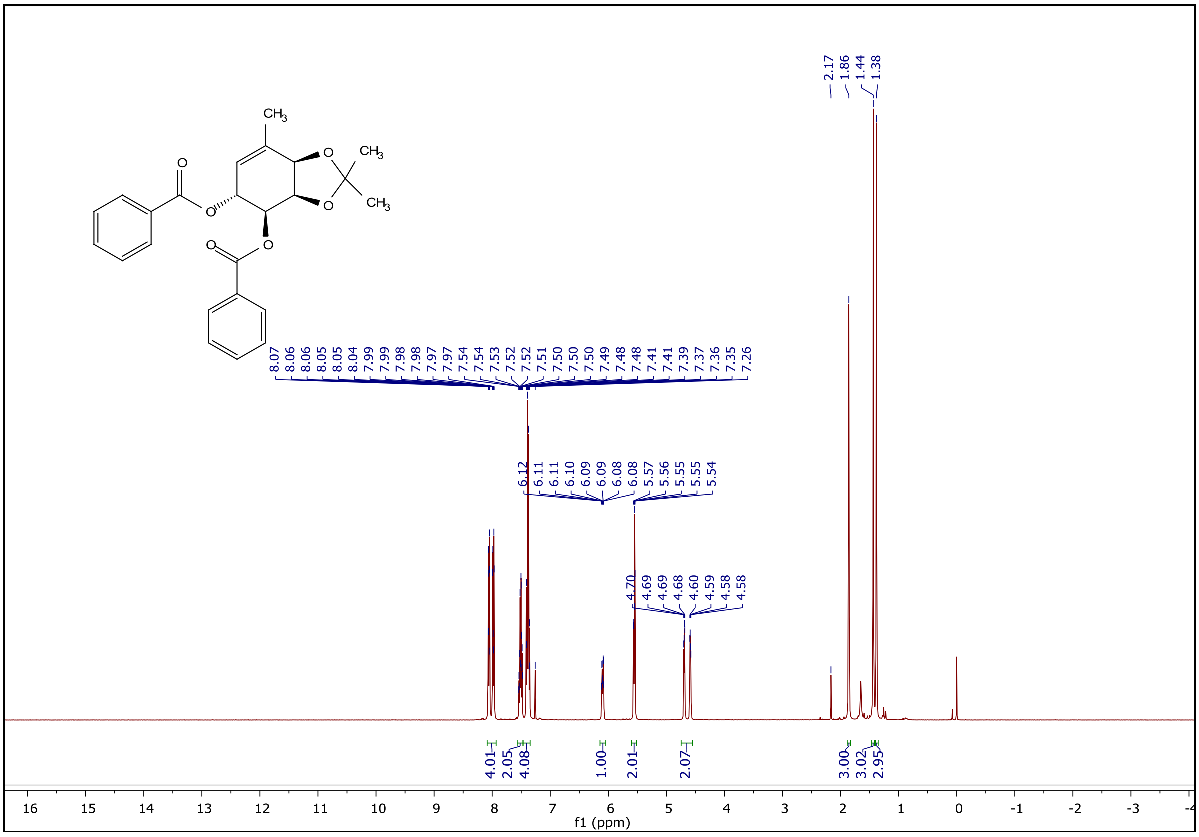

Figure S2. Extended ^{1}H-NMR spectrum of **11 (I)**

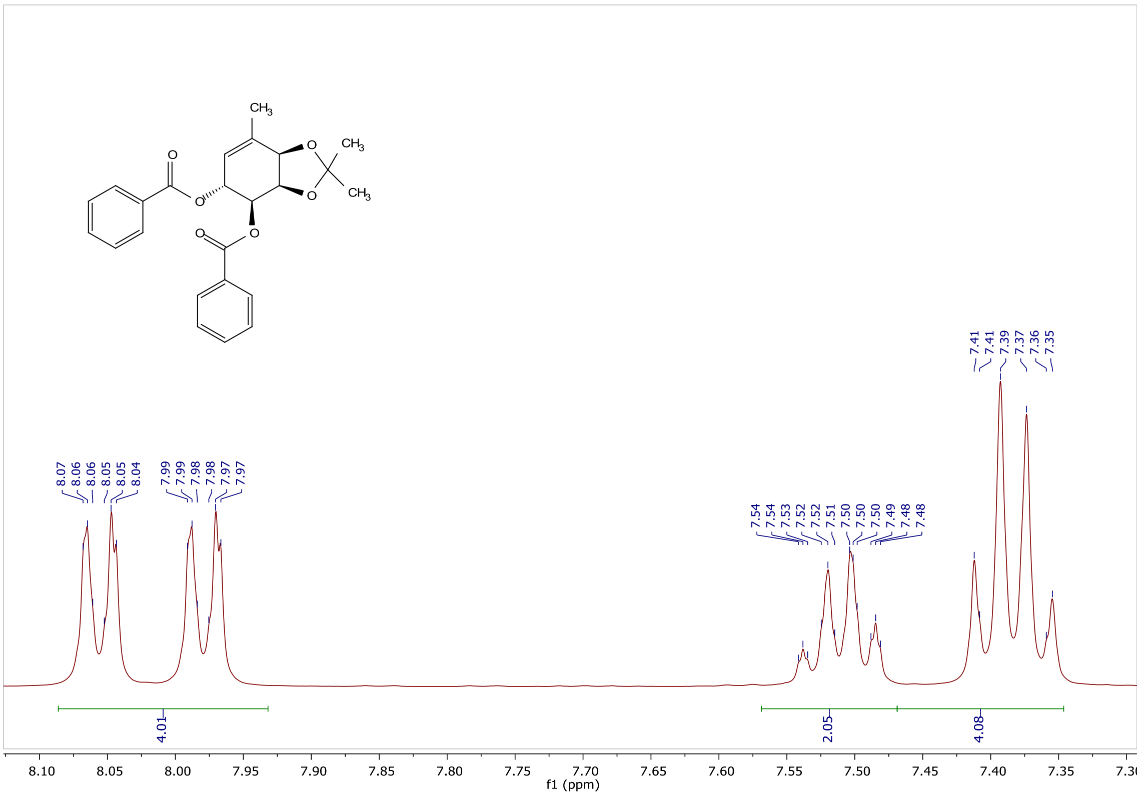

Figure S3. Extended ^{1}H-NMR spectrum of **11 (II)**

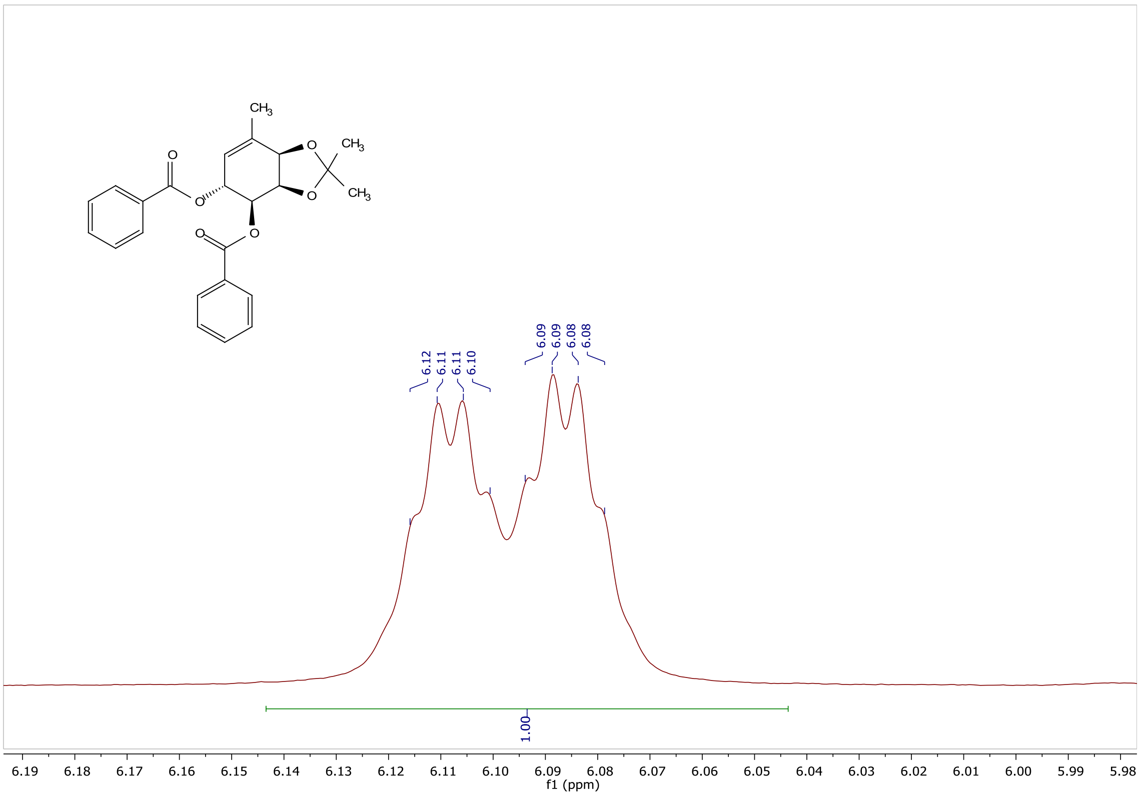

Figure S4. Extended ^{1}H-NMR spectrum of **11 (III)**

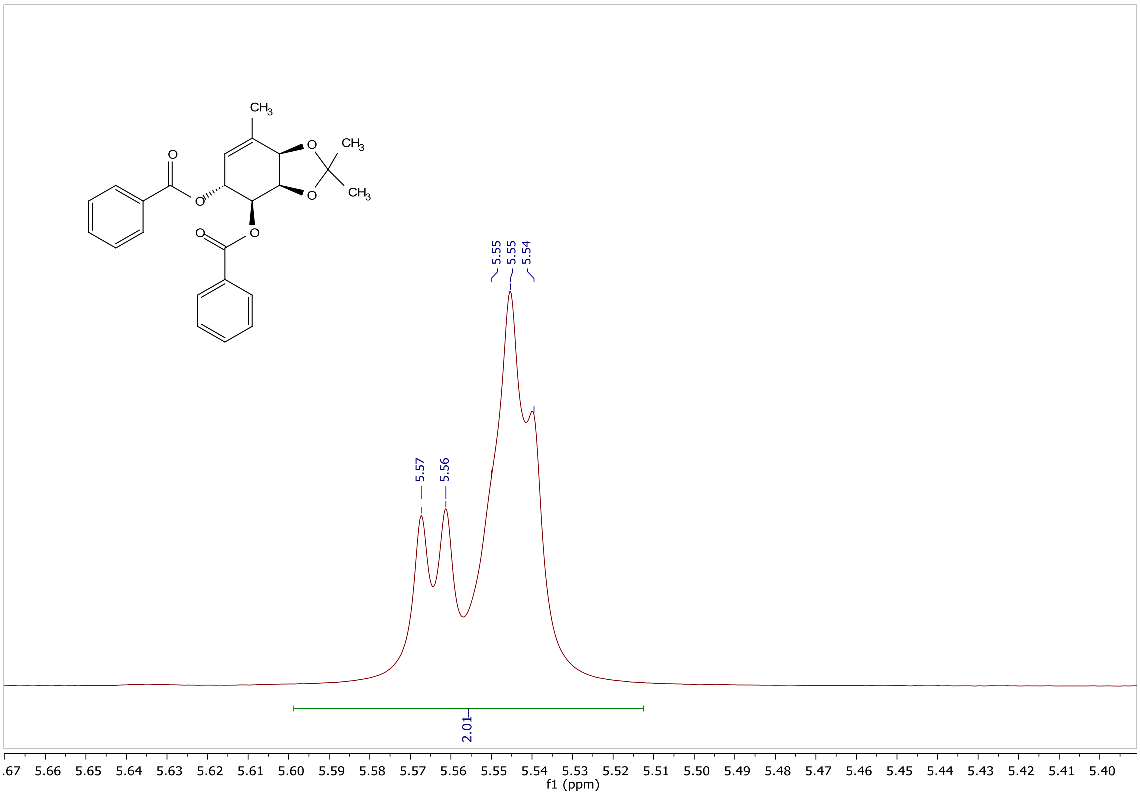

Figure S5. Extended ^{1}H-NMR spectrum of **11 (IV)**

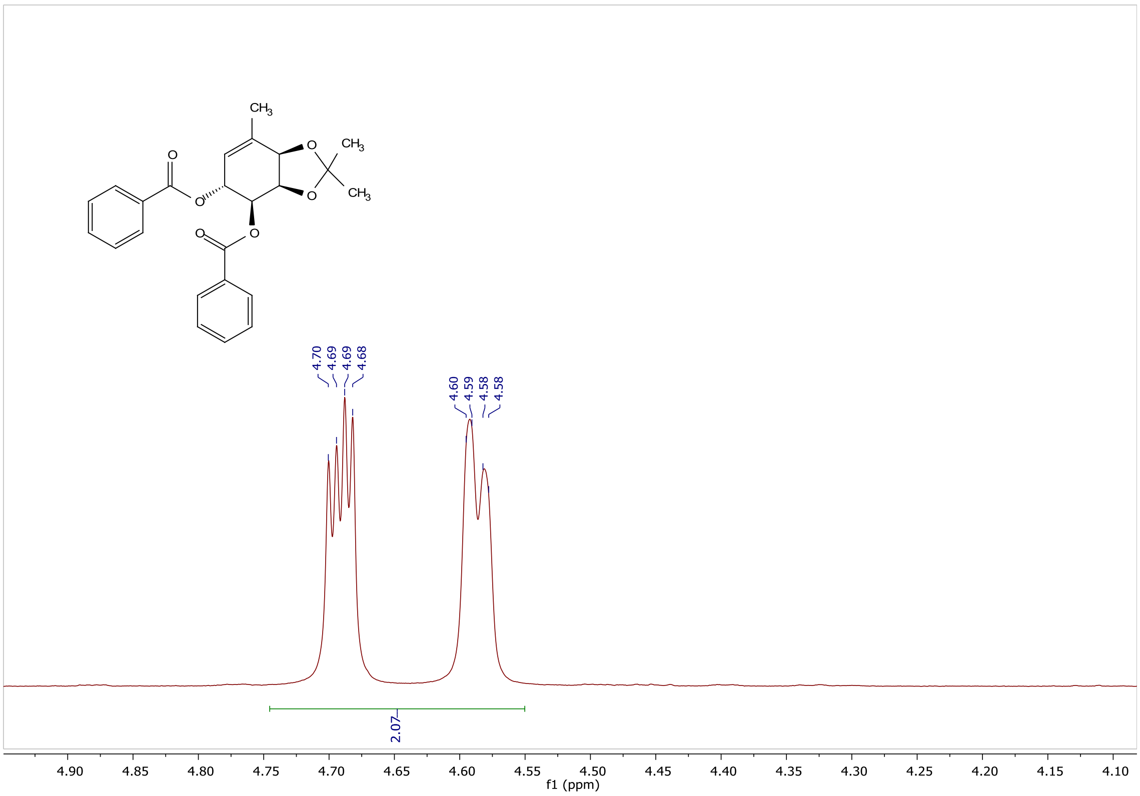

Figure S6. Extended ^{1}H-NMR spectrum of **11 (V)**

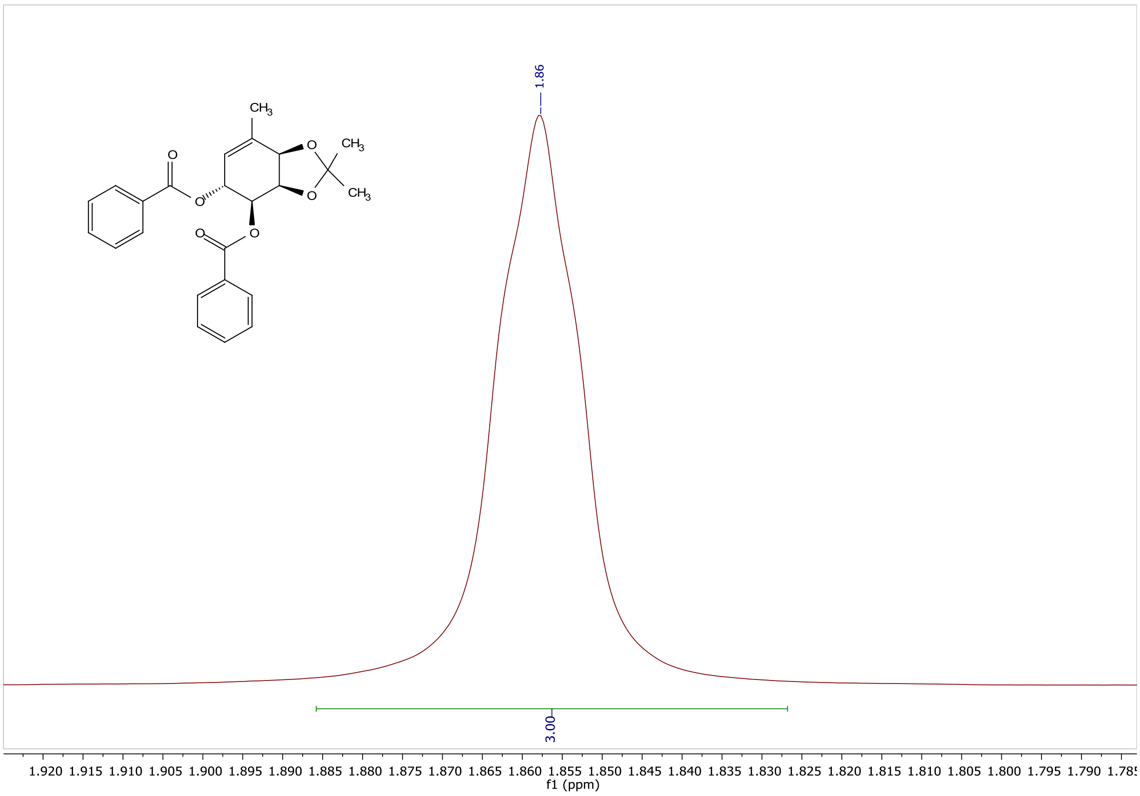

Figure S7. Extended ^{1}H-NMR spectrum of **11 (VI)**

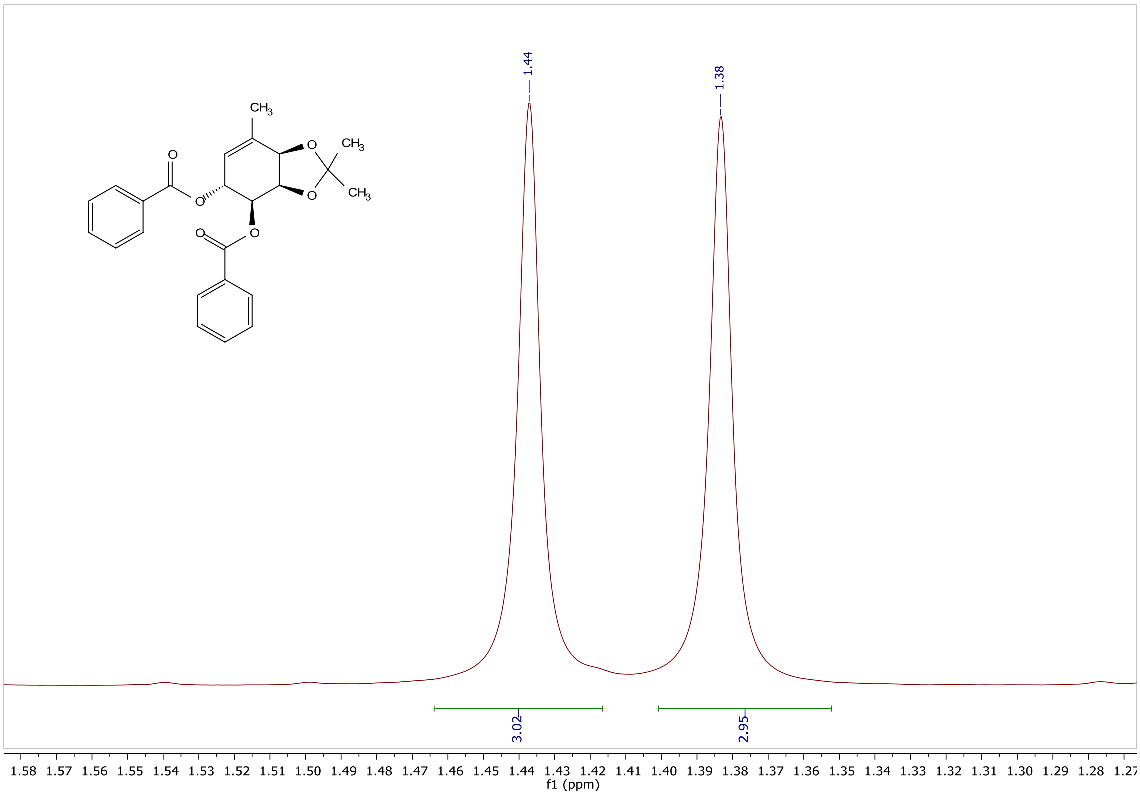

Figure S8. ^{13}C-NMR spectrum of **11**

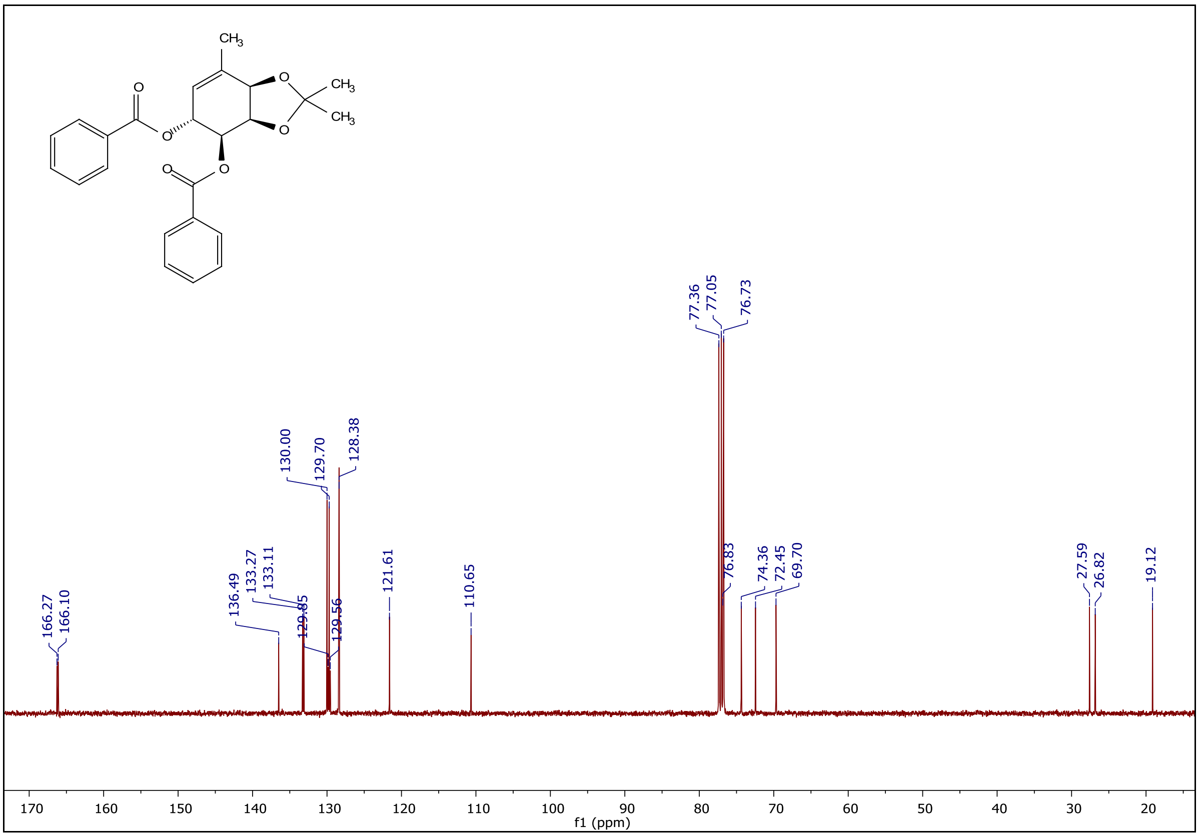

Figure S9. IR spectrum of **11**

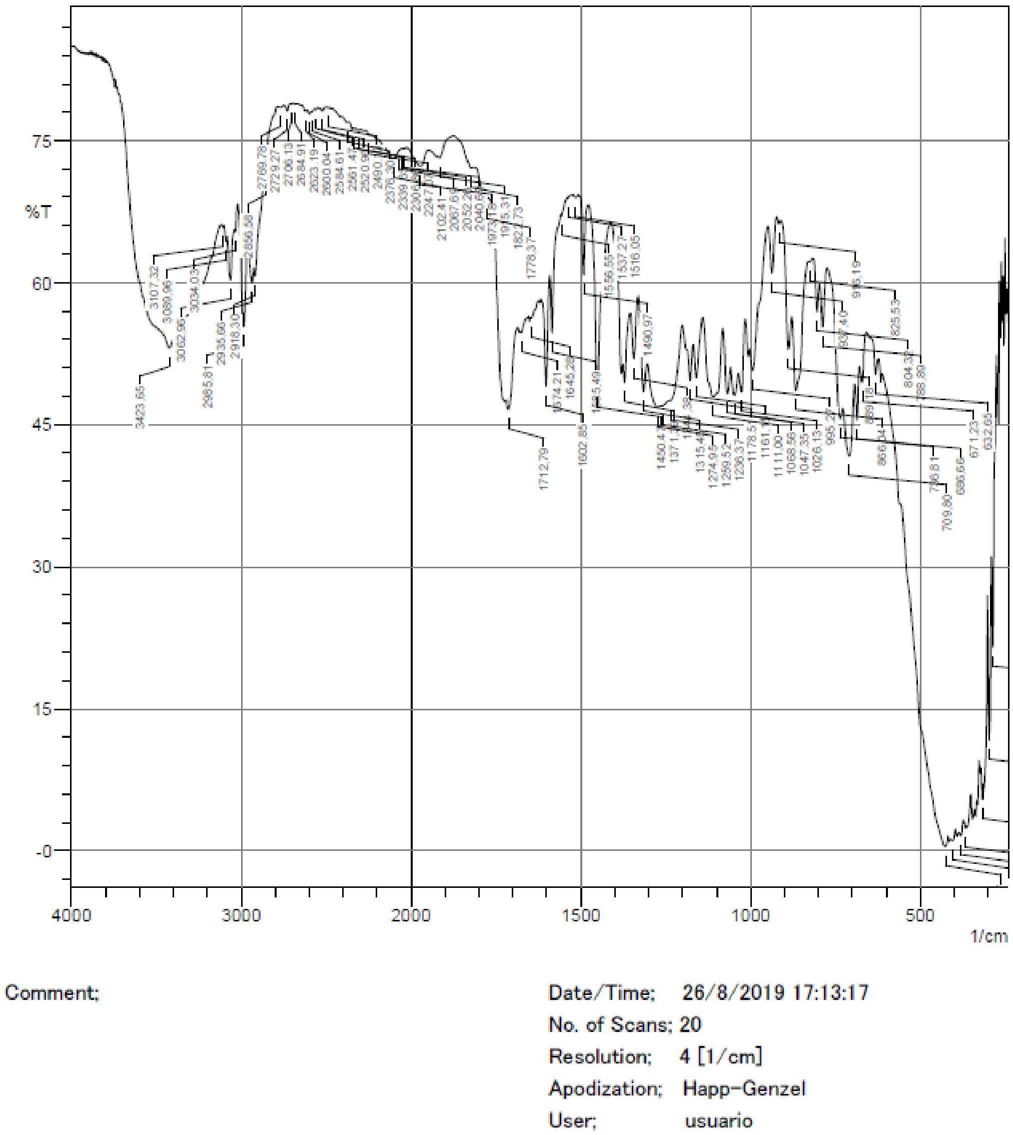

2) *(1R,2R,5R,6R)-5,6-dibenzoyl-3-methylciclohex-3-en-1,2,5,6-tetraol* (**12**)

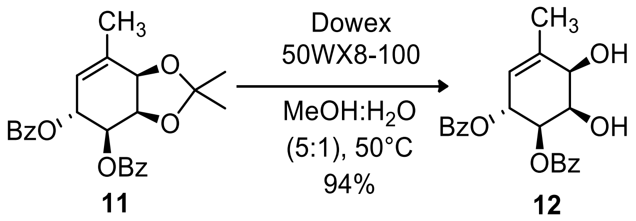

Figure S10. ^{1}H-NMR spectrum of **12**

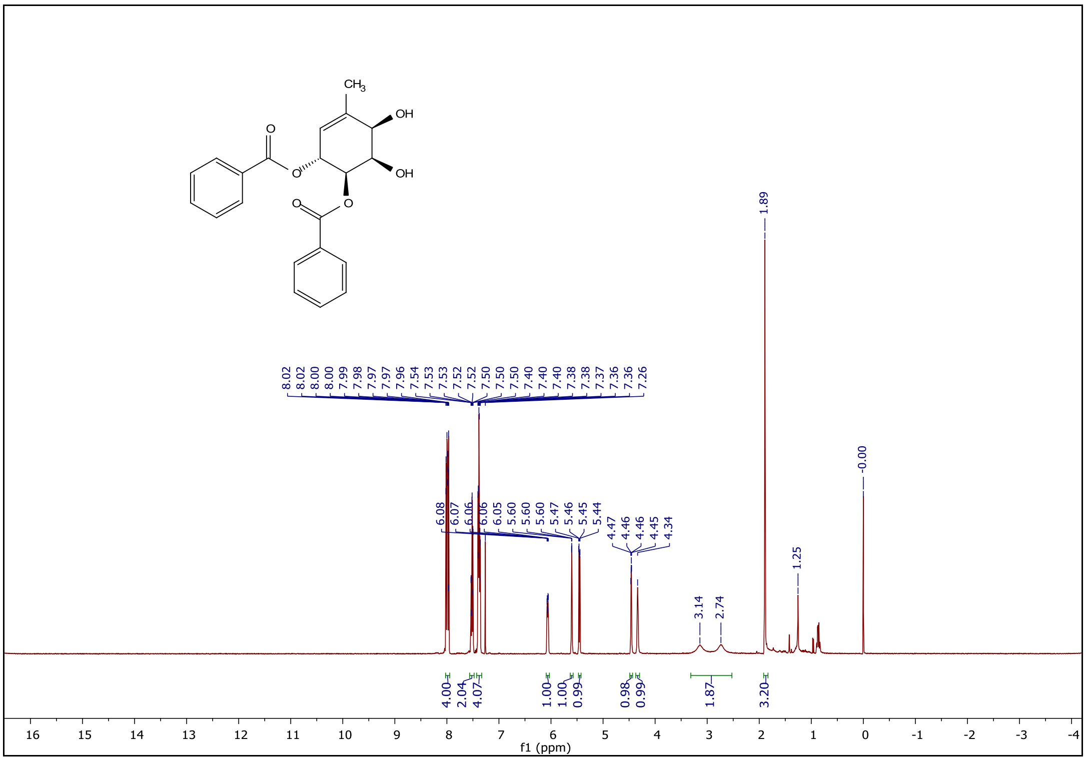

Figure S11. Extended ^{1}H-NMR spectrum of **12 (I)**

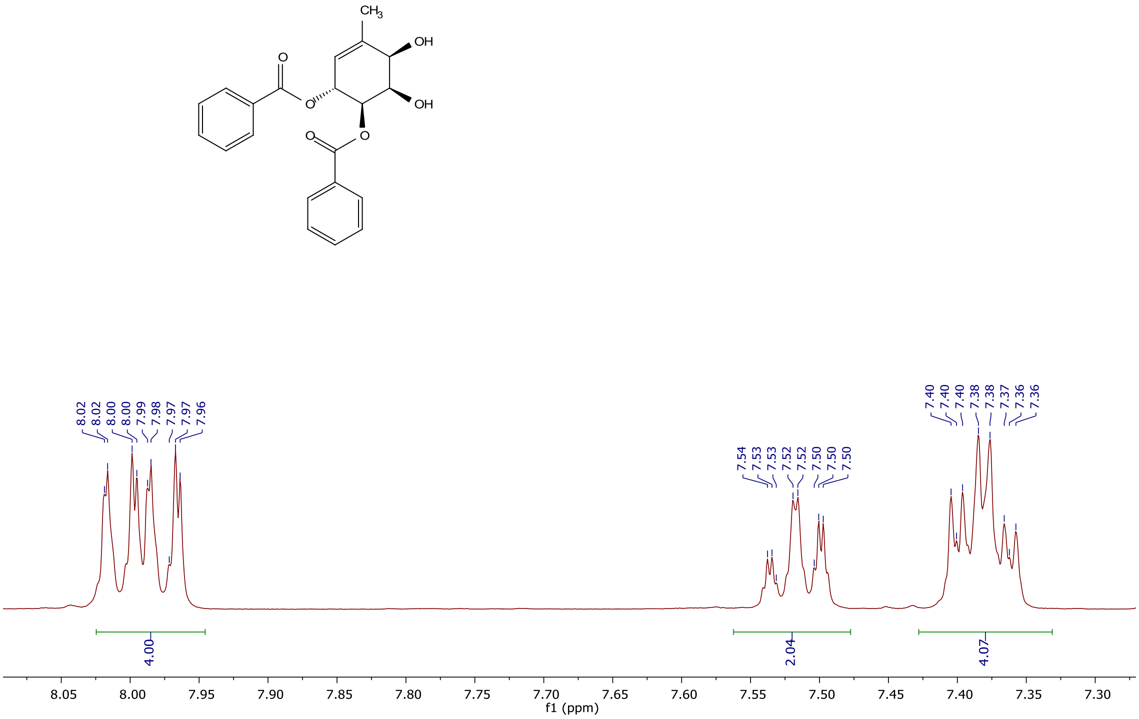

Figure S12. Extended ^{1}H-NMR spectrum of **12 (II)**

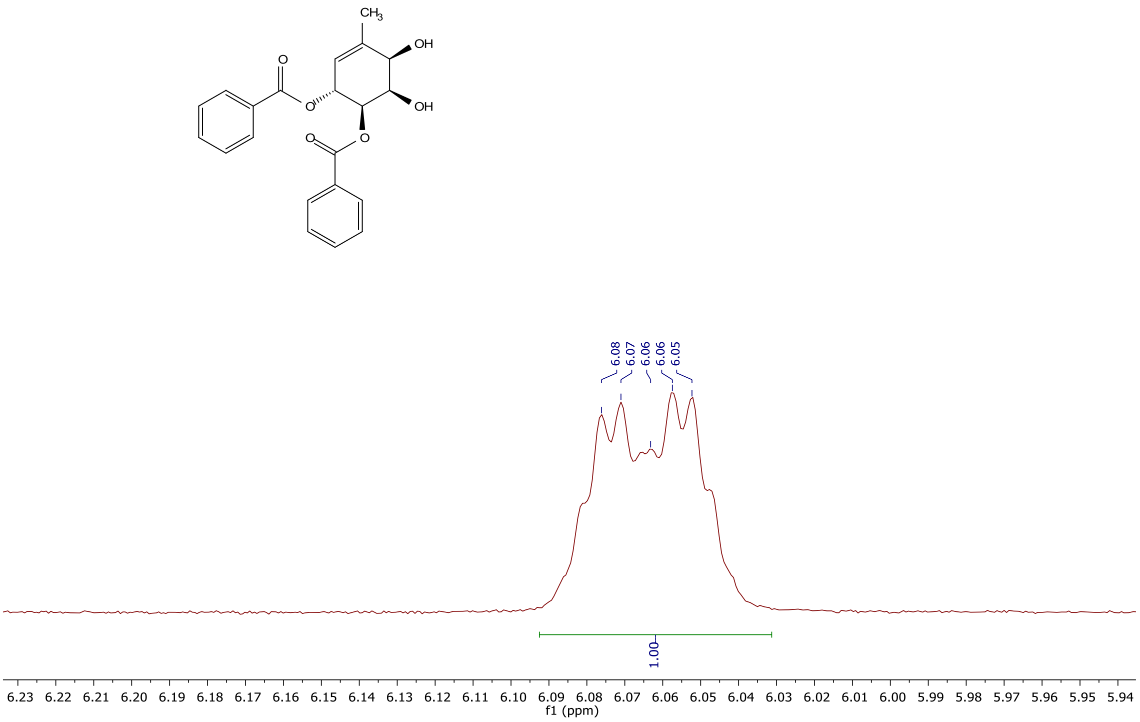

Figure S13. Extended ^{1}H-NMR spectrum of **12 (III)**

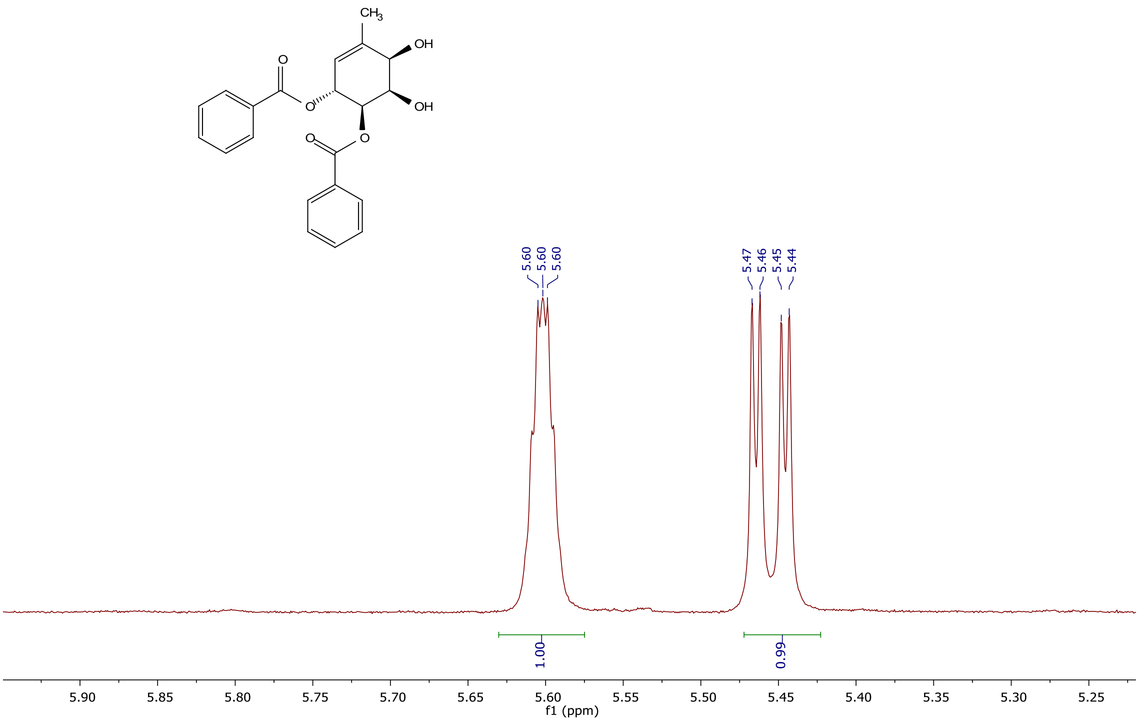

Figure S14. Extended ^{1}H-NMR spectrum of **12 (IV)**

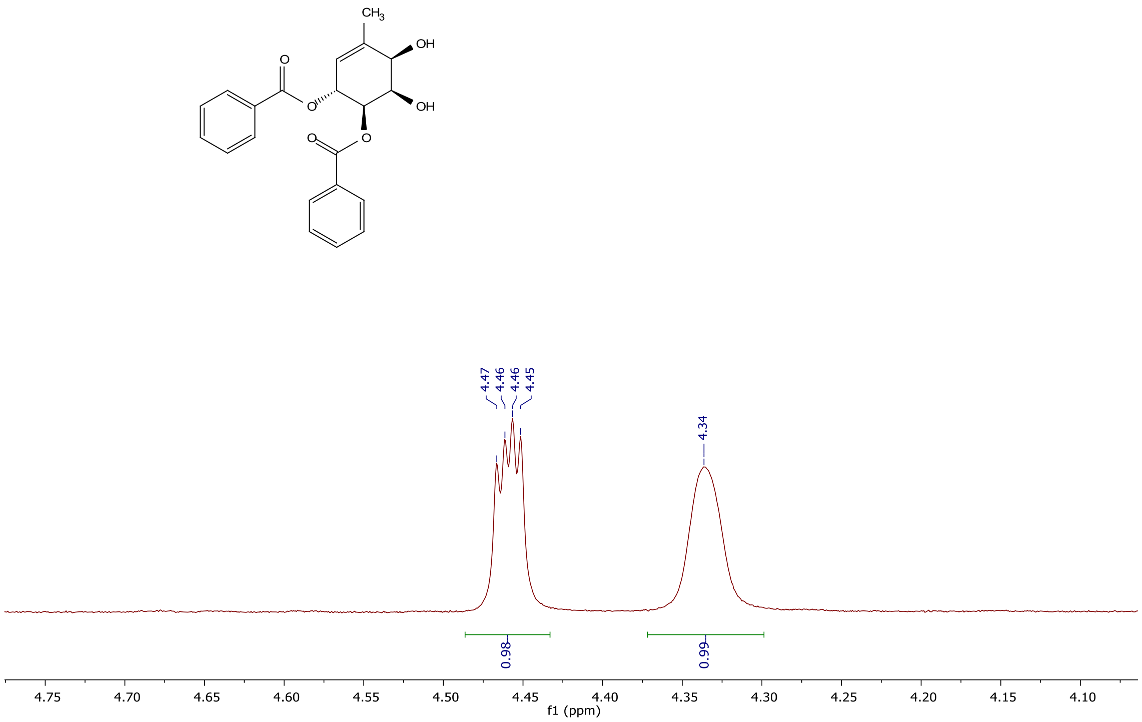

Figure S15. Extended ^{1}H-NMR spectrum of **12 (V)**

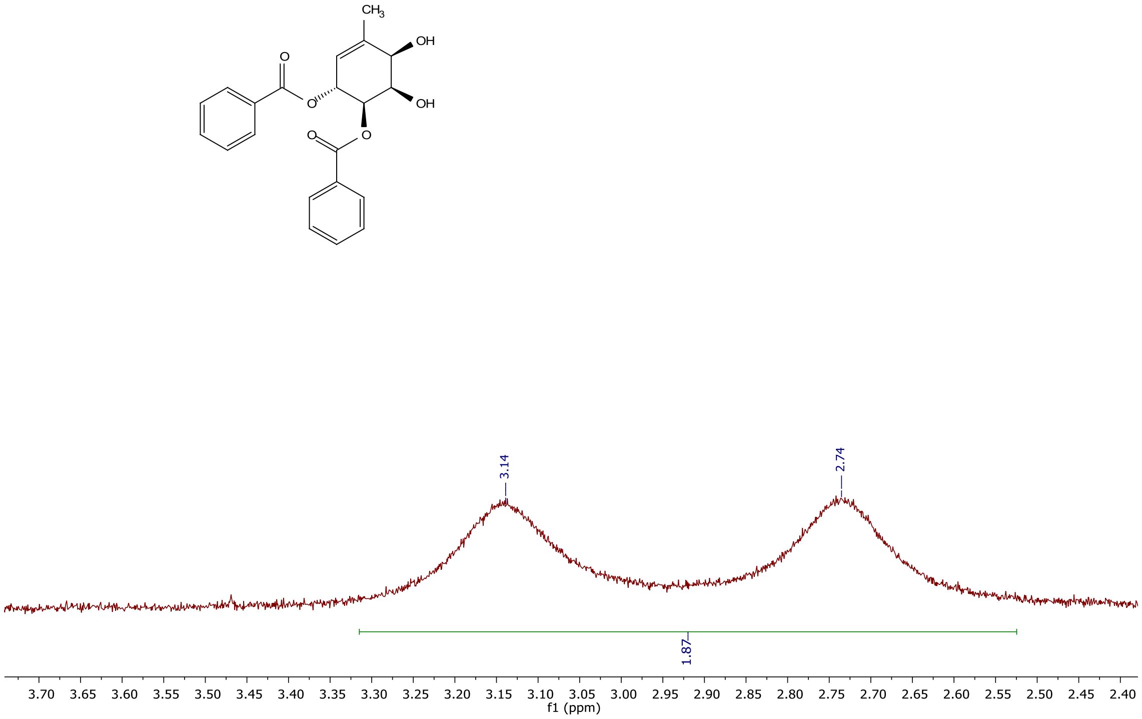

Figure S16. Extended ^{1}H-NMR spectrum of **12 (VI)**

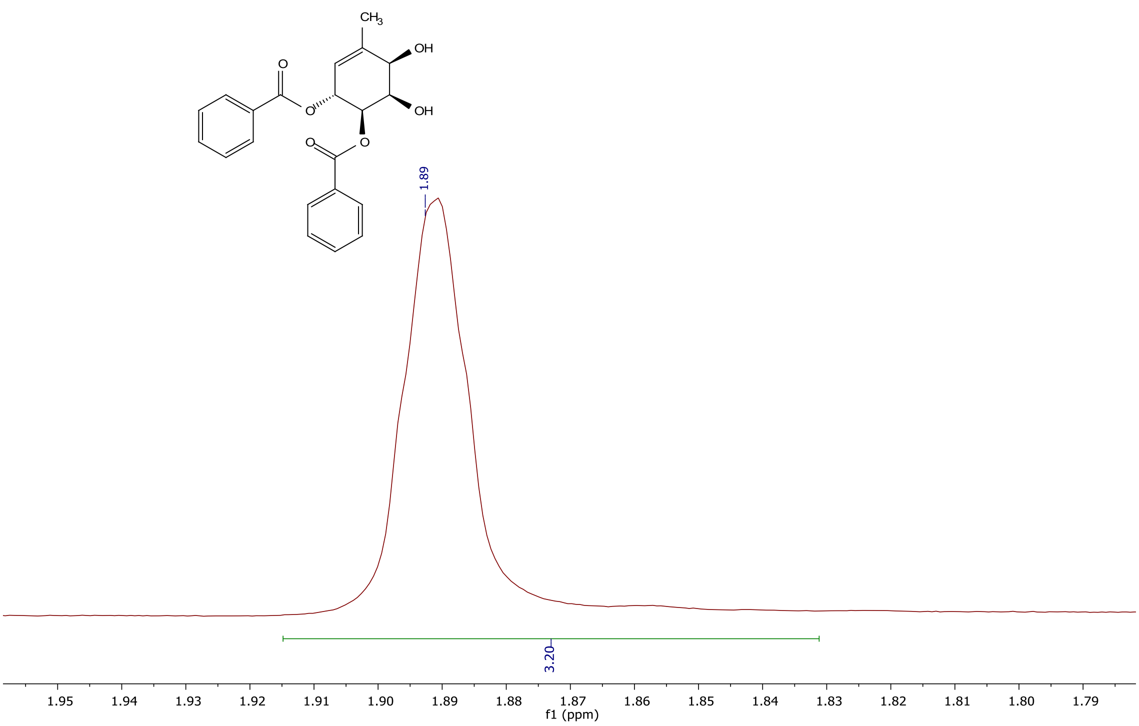

Figure S17. ^{13}C-NMR spectrum of **12**

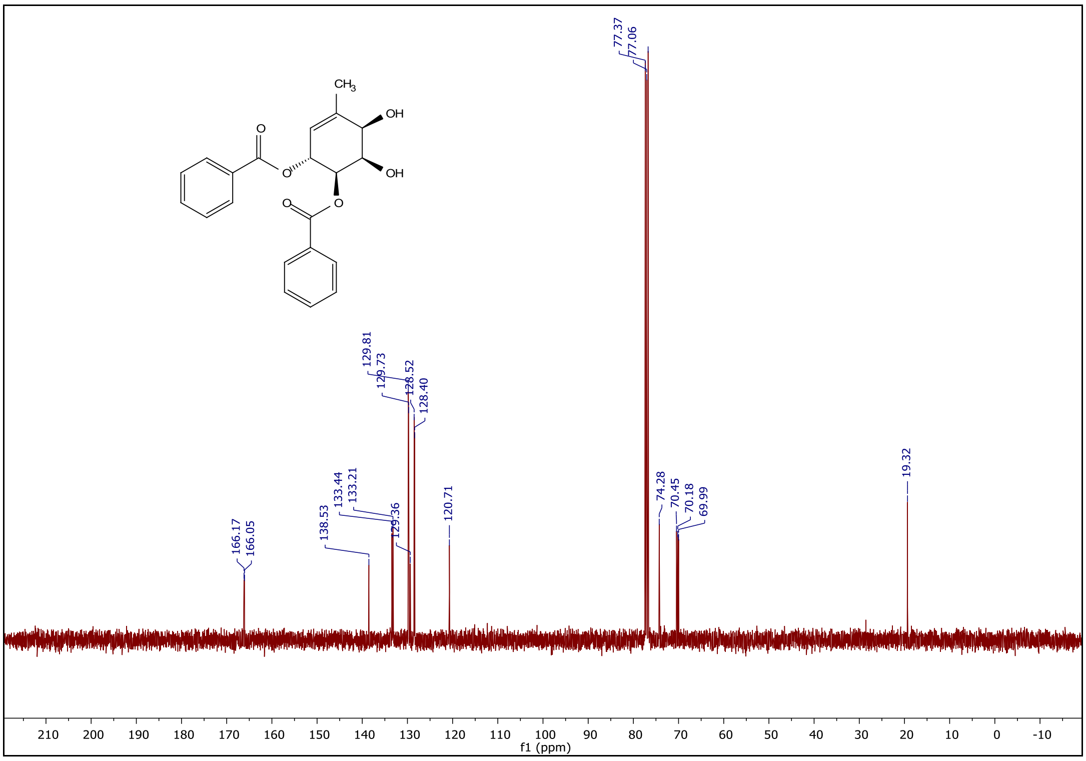

Figure S18. IR spectrum of **12**

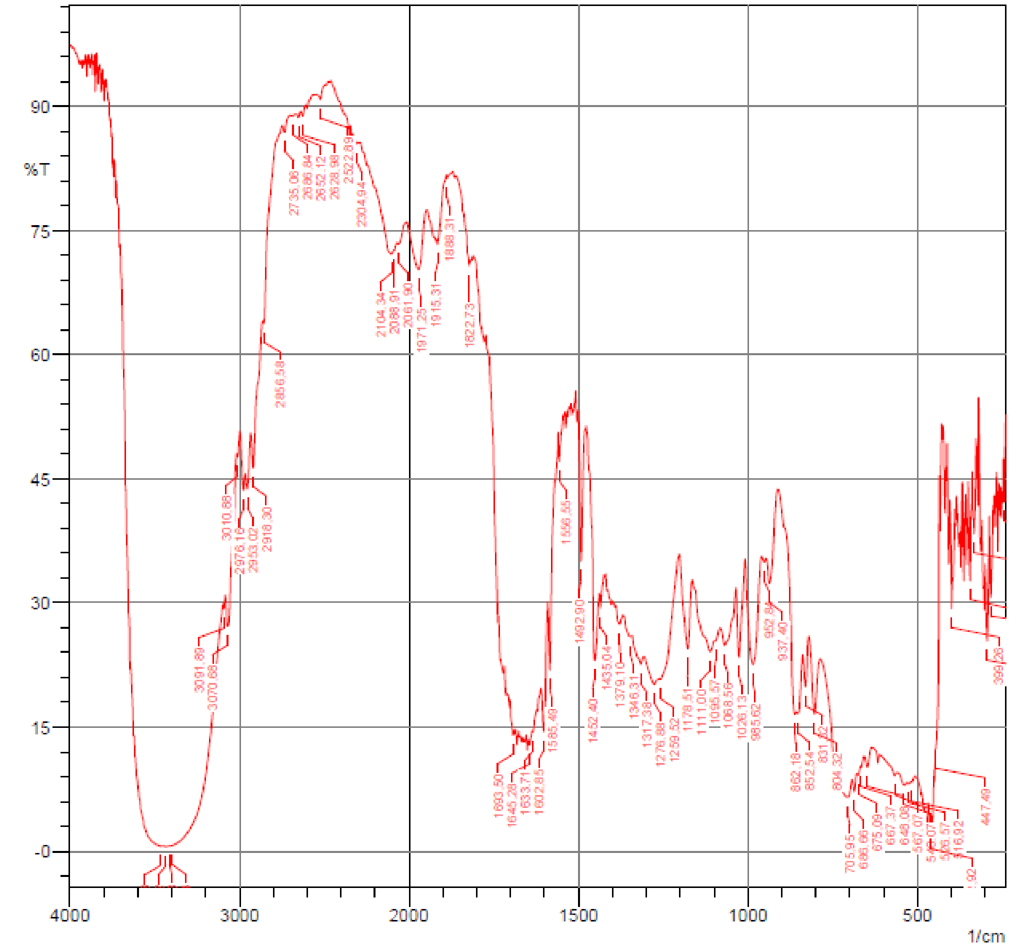

Comment;

Date/Time; 26/8/2019 18:52:25
No. of Scans; 20
Resolution; 4 [1/cm]
Apodization; Happ-Genzel
User; usuario

3) *(4R,5R,6S)-4,5-dibenzoyl-6-hydroxy-2-methylciclohex-2-enone* (**13**)

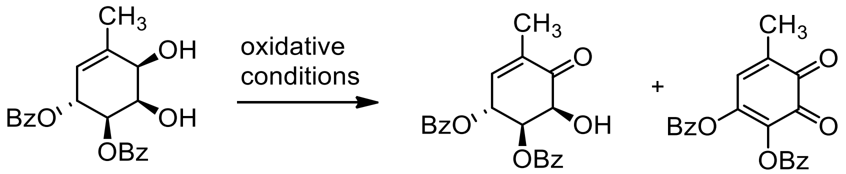

12 **13** **14**

Figure S19. ¹H-NMR spectrum of **13**

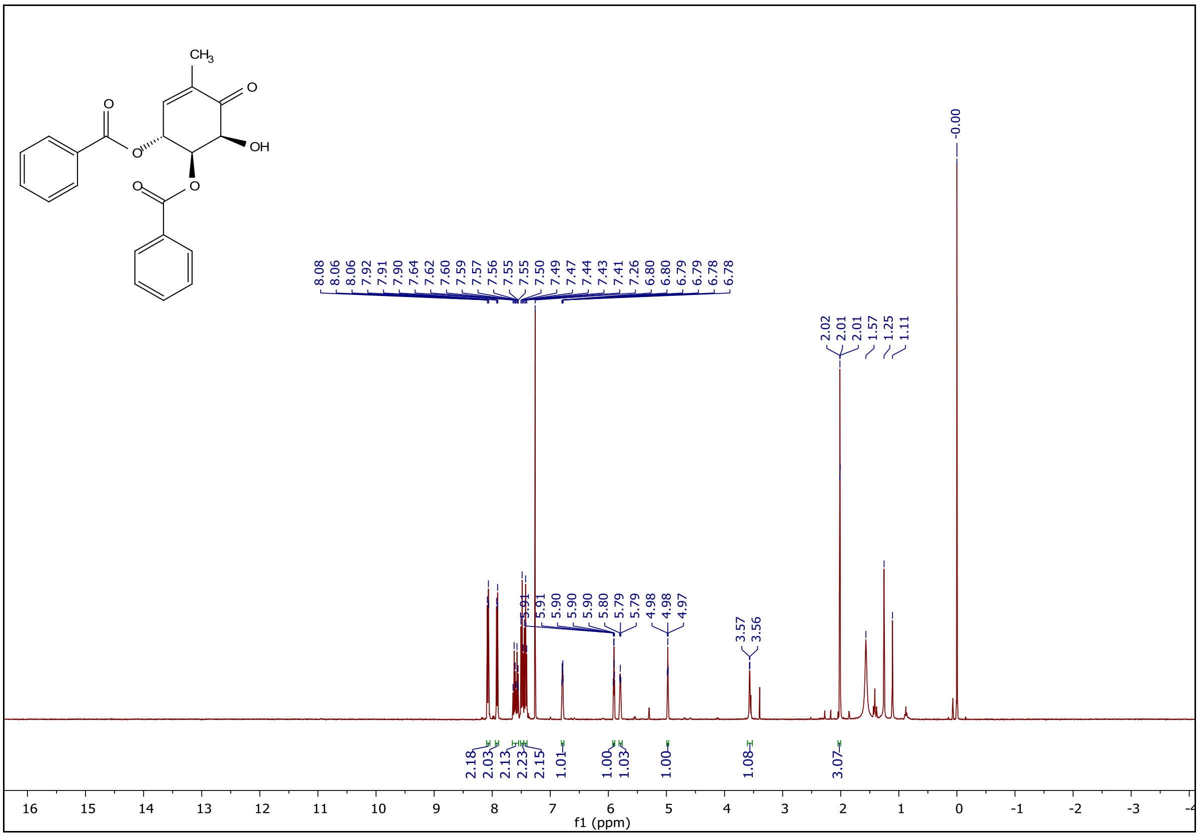

Figure S20. Extended ¹H-NMR spectrum of **13 (I)**

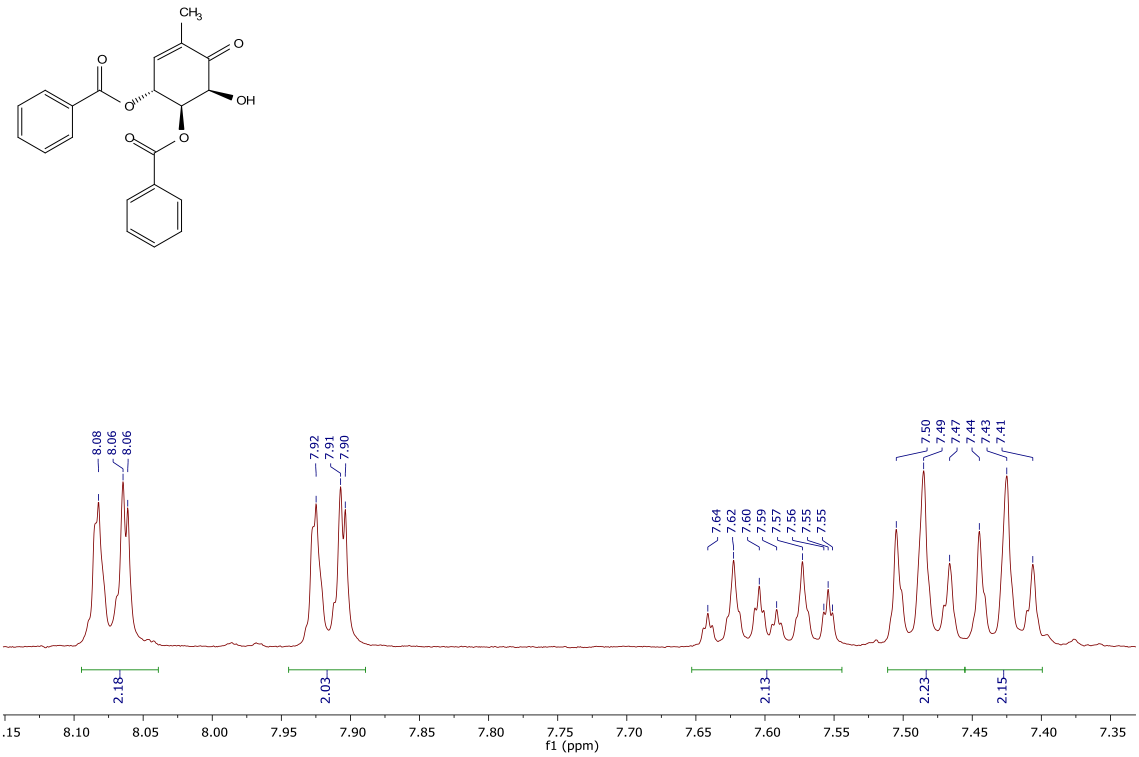

Figure S21. Extended ¹H-NMR spectrum of **13 (II)**

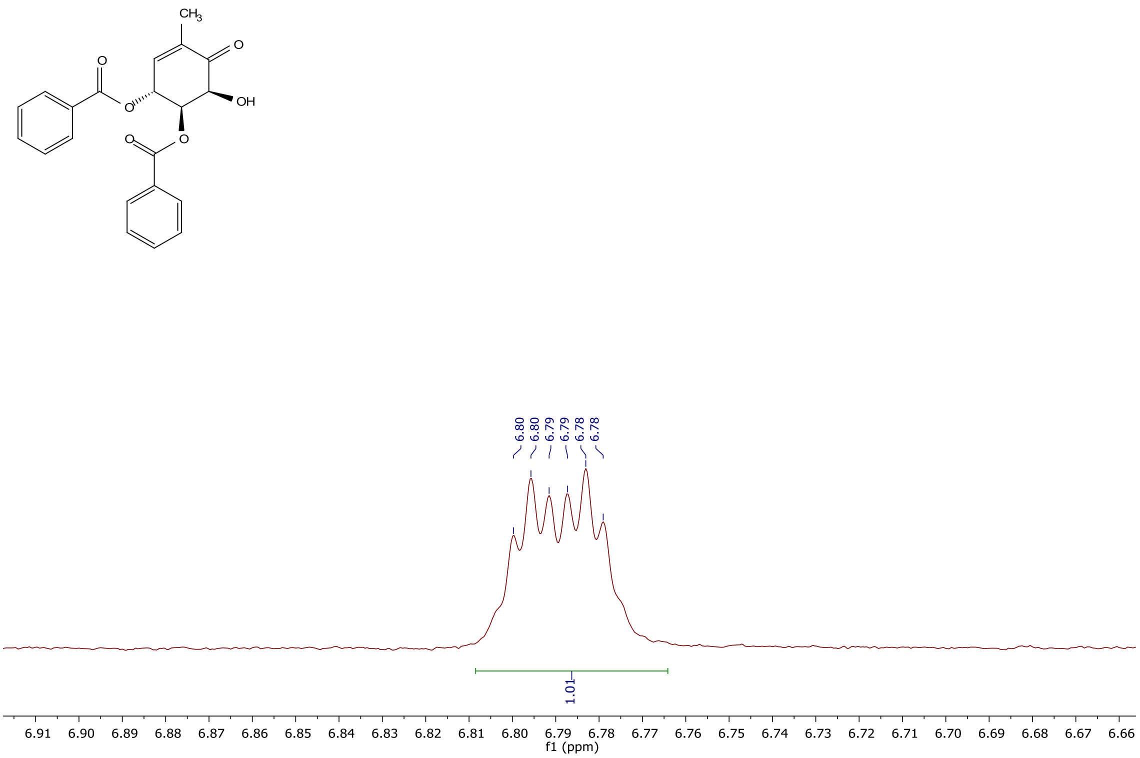

Figure S22. Extended ^{1}H-NMR spectrum of **13 (III)**

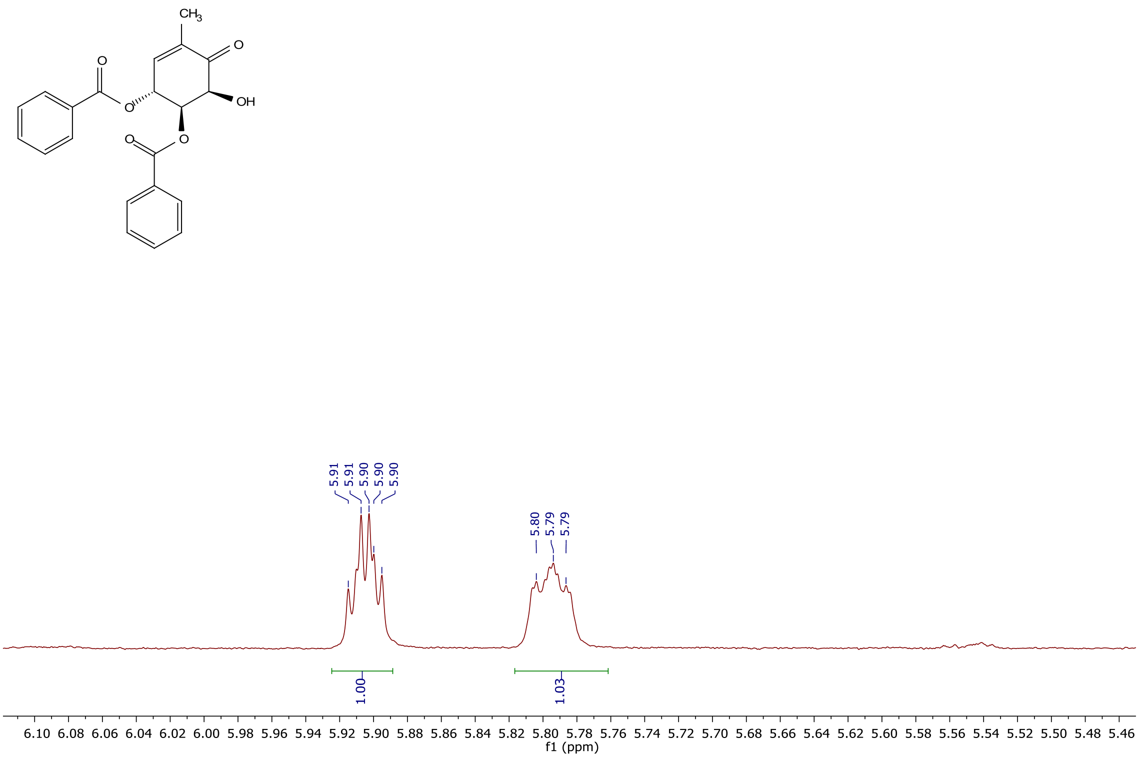

Figure S23. Extended ^{1}H-NMR spectrum of **13 (IV)**

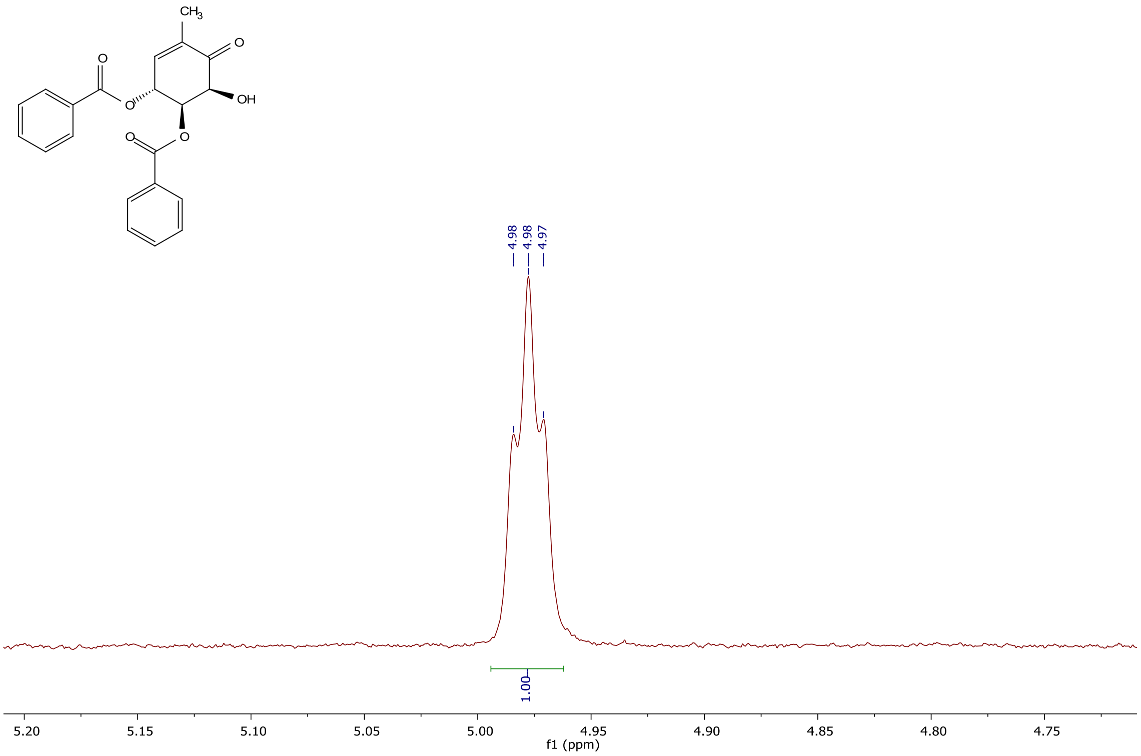

Figure S24. Extended ^{1}H-NMR spectrum of **13 (V)**

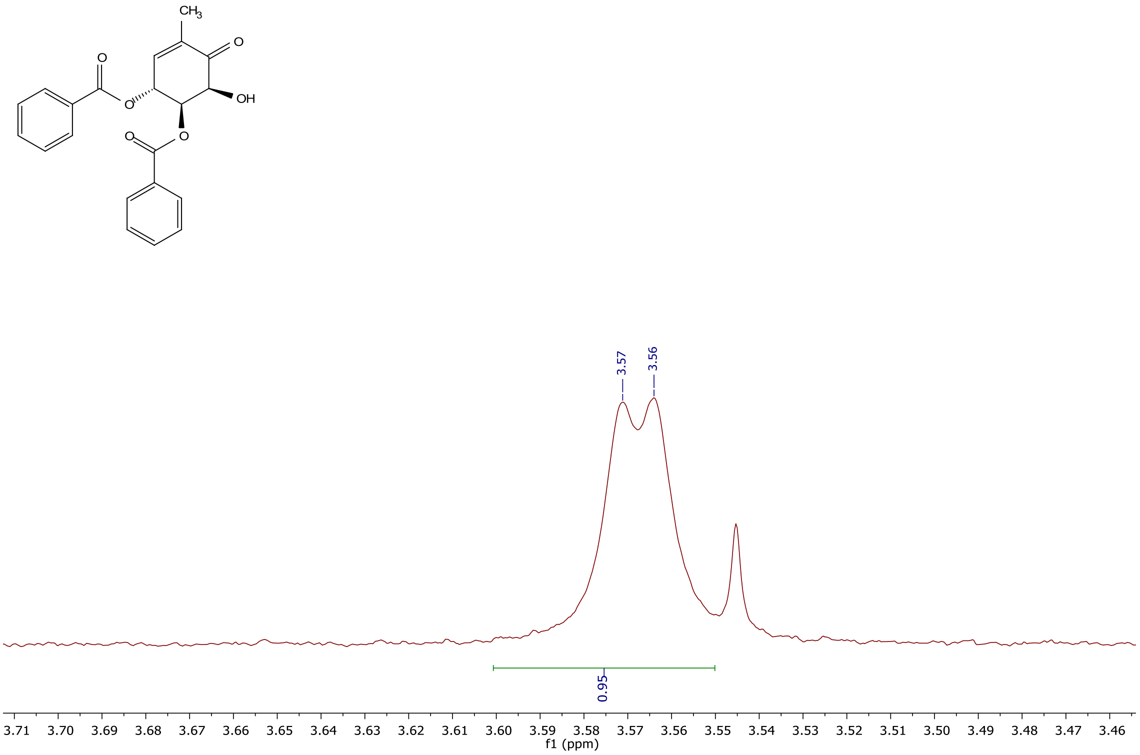

Figure S25. Extended ^{1}H-NMR spectrum of **13 (VI)**

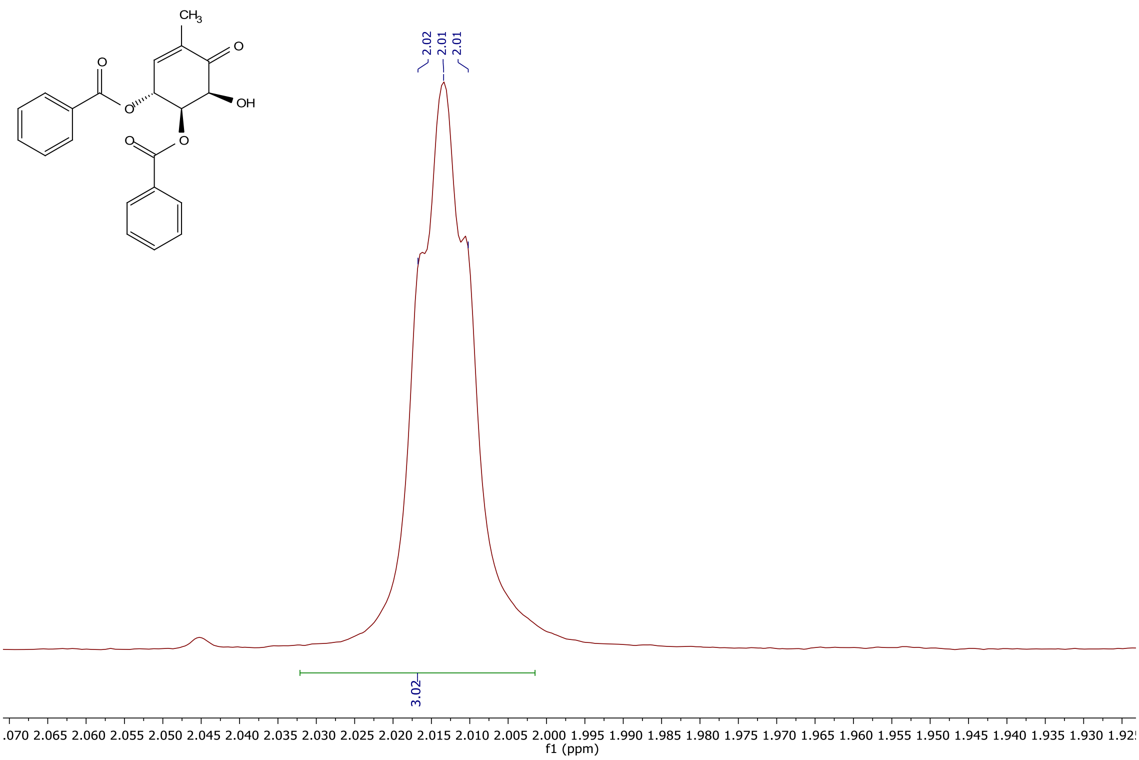

Figure S26. ^{13}C-NMR of **13**

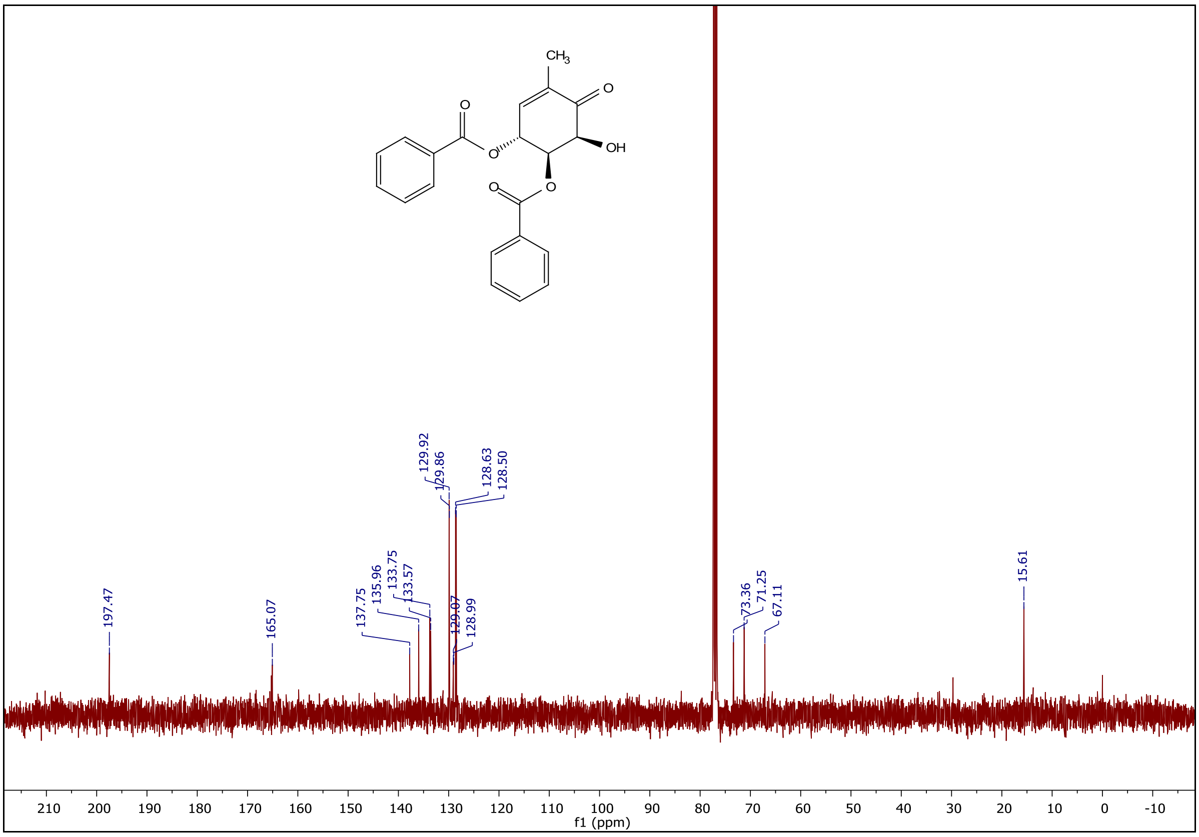

Figure S27. IR spectrum of **13**

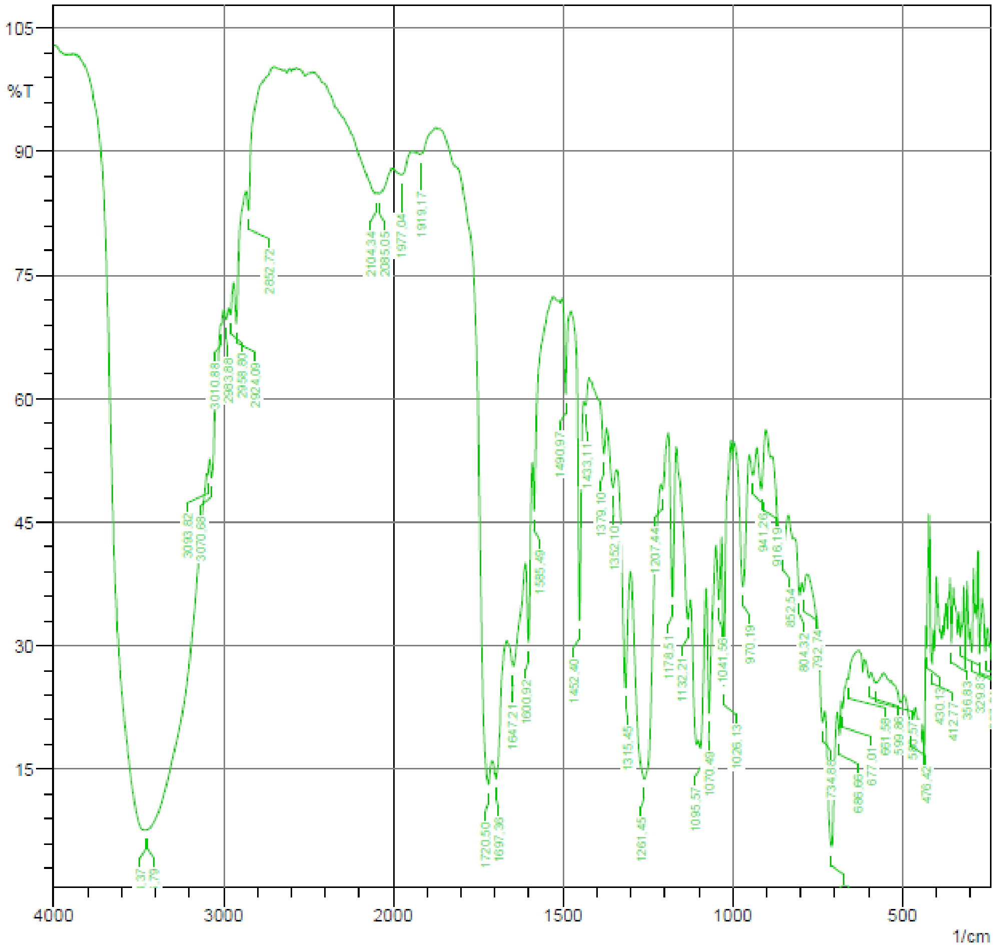

Comment;

Date/Time; 26/8/2019 19:11:46
No. of Scans; 20
Resolution; 4 [1/cm]
Apodization; Happ-Genzel
User; usuario

Figure S28. ^{1}H-NMR spectrum of **14**

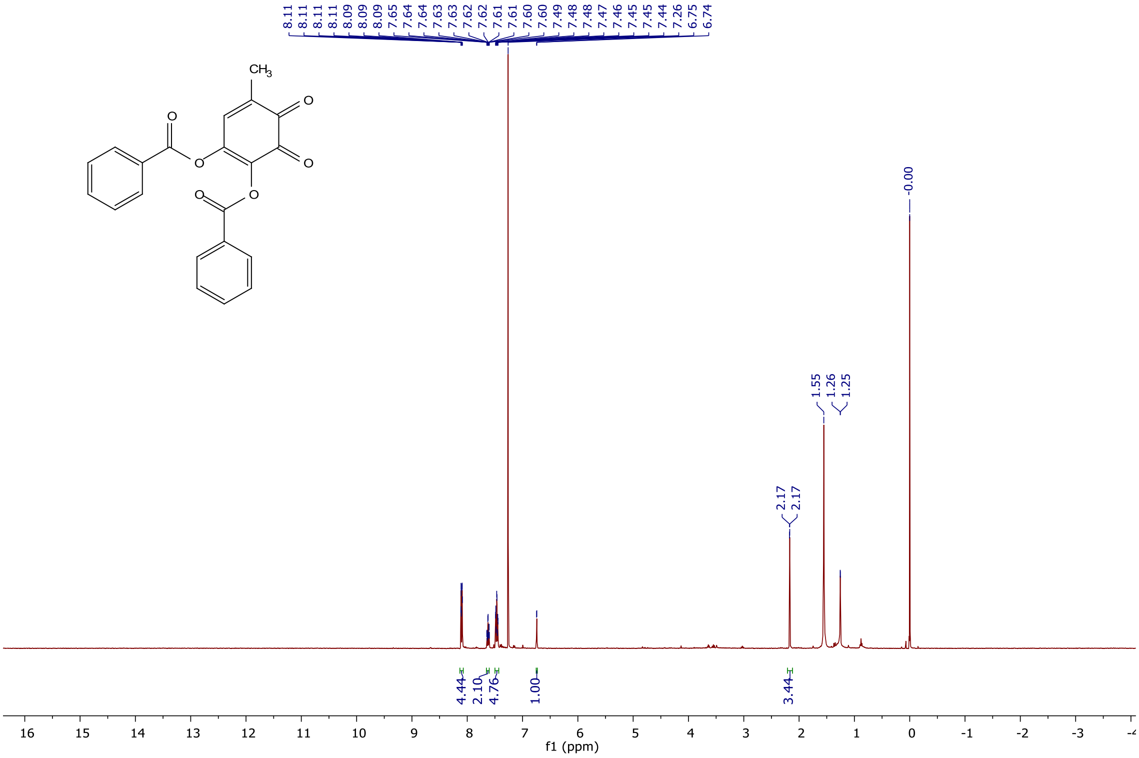

Figure S29. COSY experiment of **14**

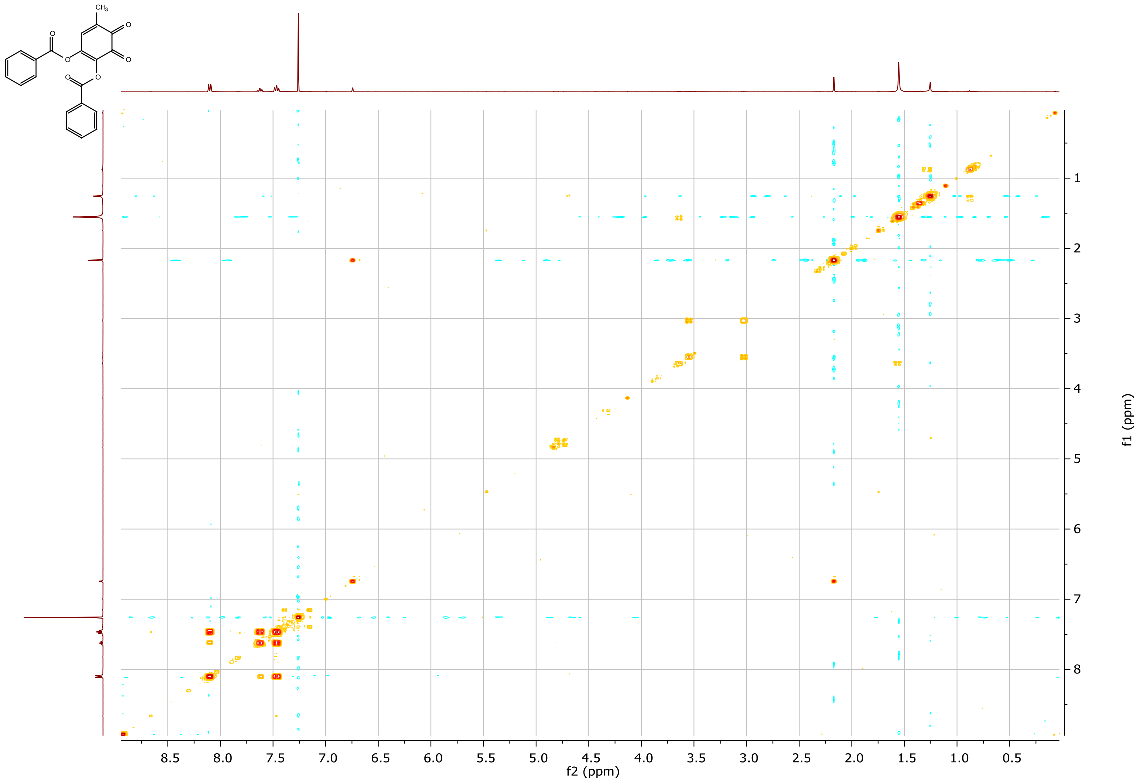

4) *(4R,5S,6S)-4,5,6-trihidroxy-2-methylcyclohex-2-enone* (NNG3)

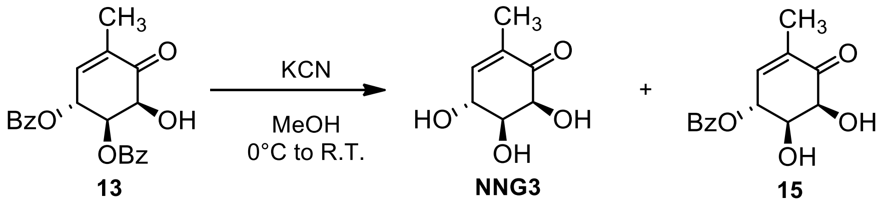

Figure S30. ^{1}H-NMR spectrum of **NNG3**

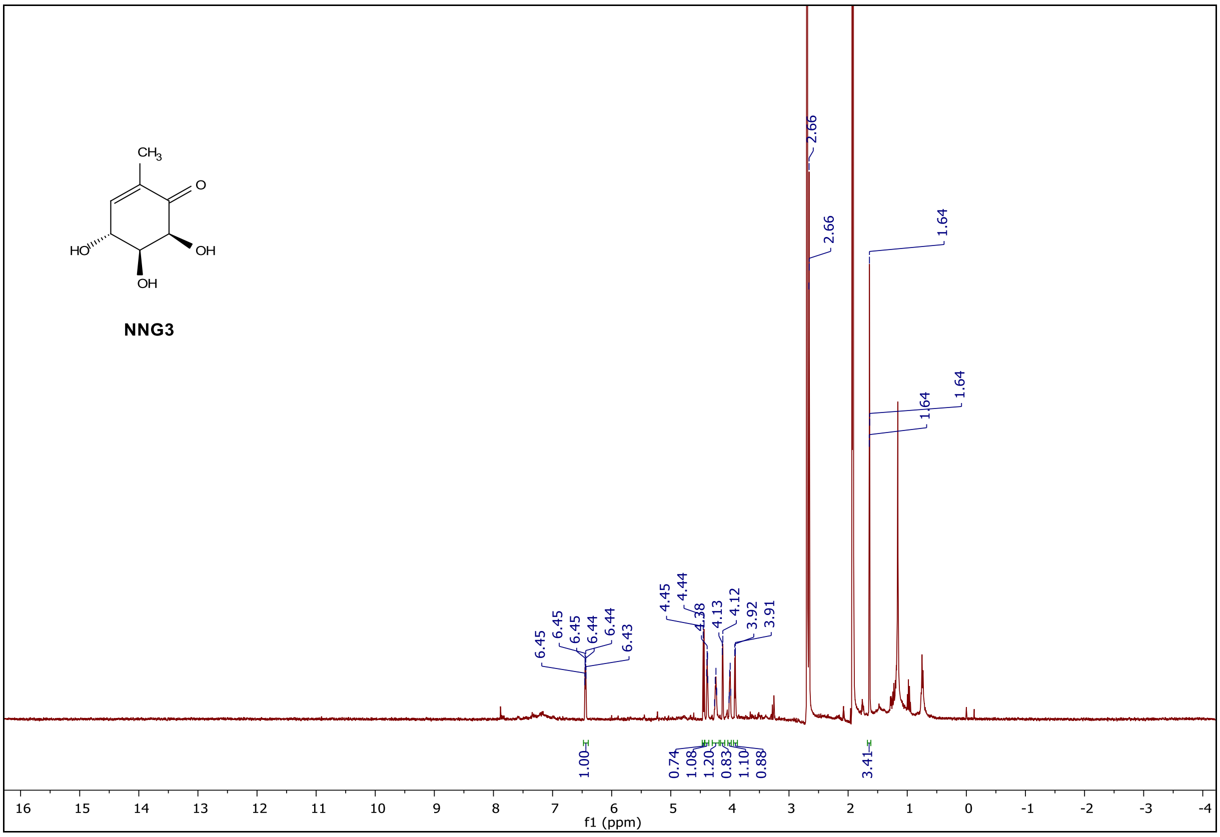

Figure S31. Extended ^{1}H-NMR spectrum of **NNG3 (I)**

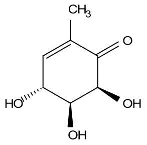

NNG3

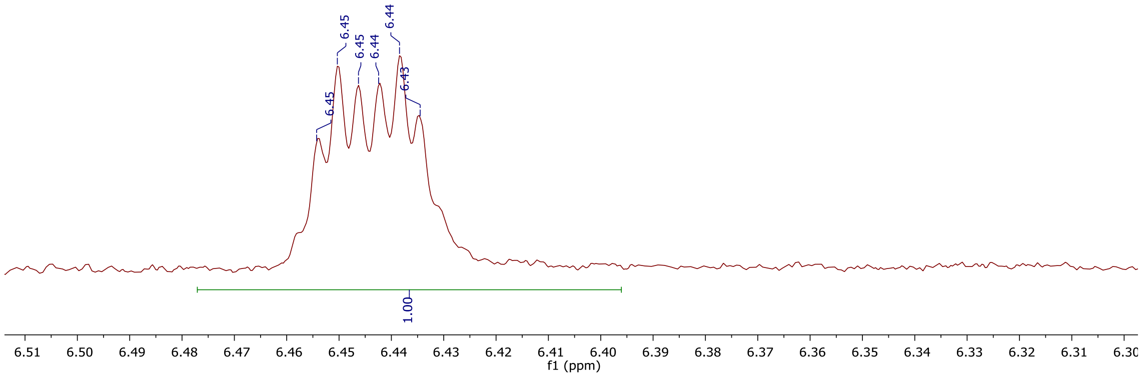

Figure S32. Extended ^{1}H-NMR spectrum of **NNG3 (II)**

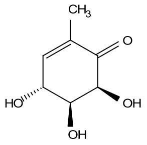

NNG3

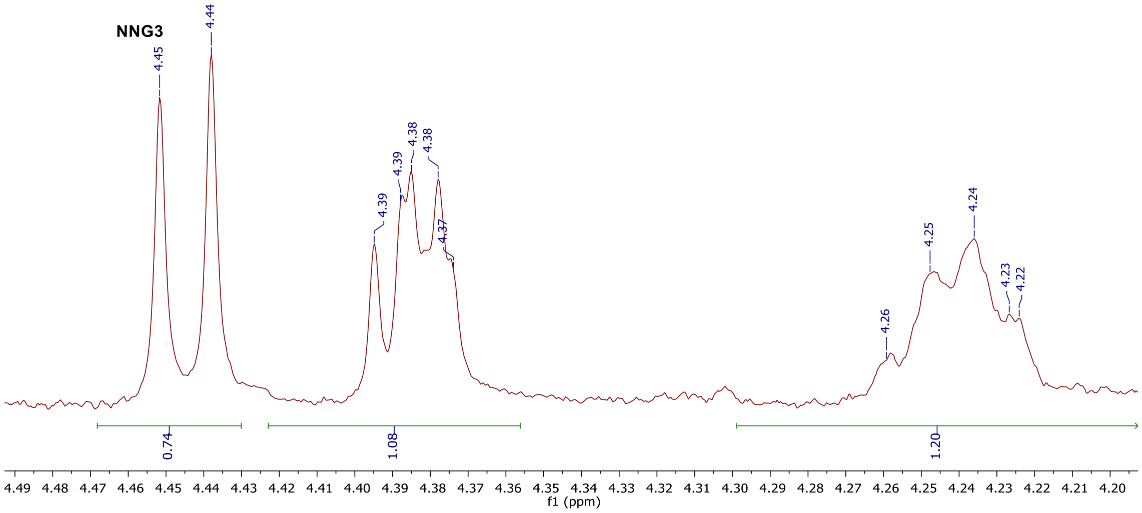

Figure S33. Extended ^{1}H-NMR spectrum of **NNG3 (III)**

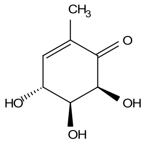

NNG3

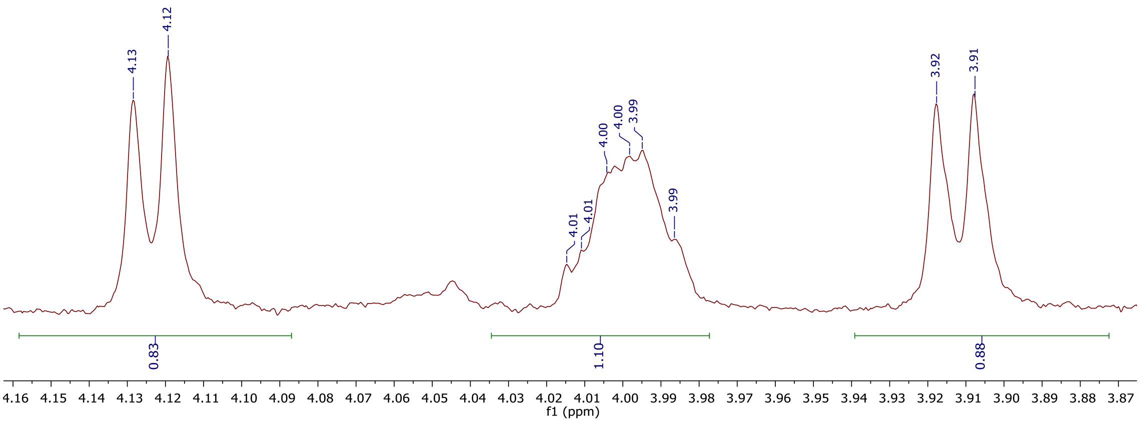

Figure S34. Extended ^{1}H-NMR spectrum of **NNG3 (IV)**

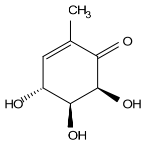

NNG3

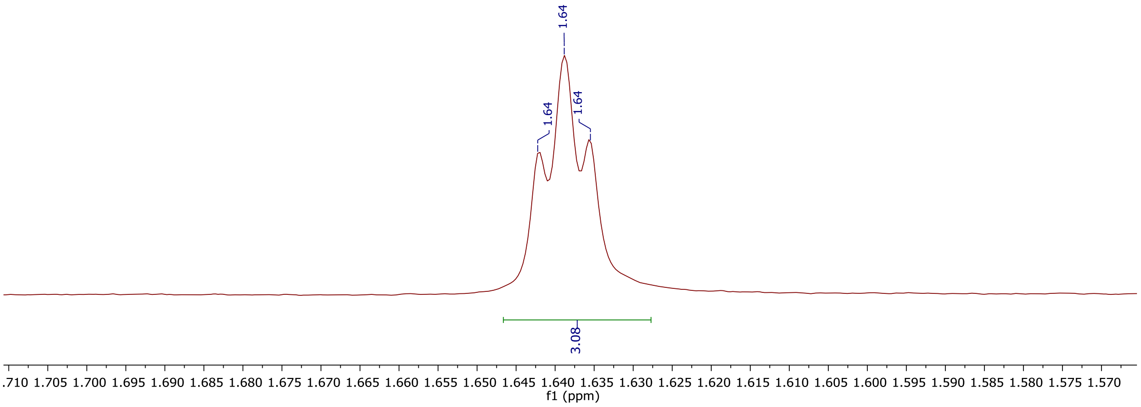

Figure S35. COSY experiment of **NNG3**

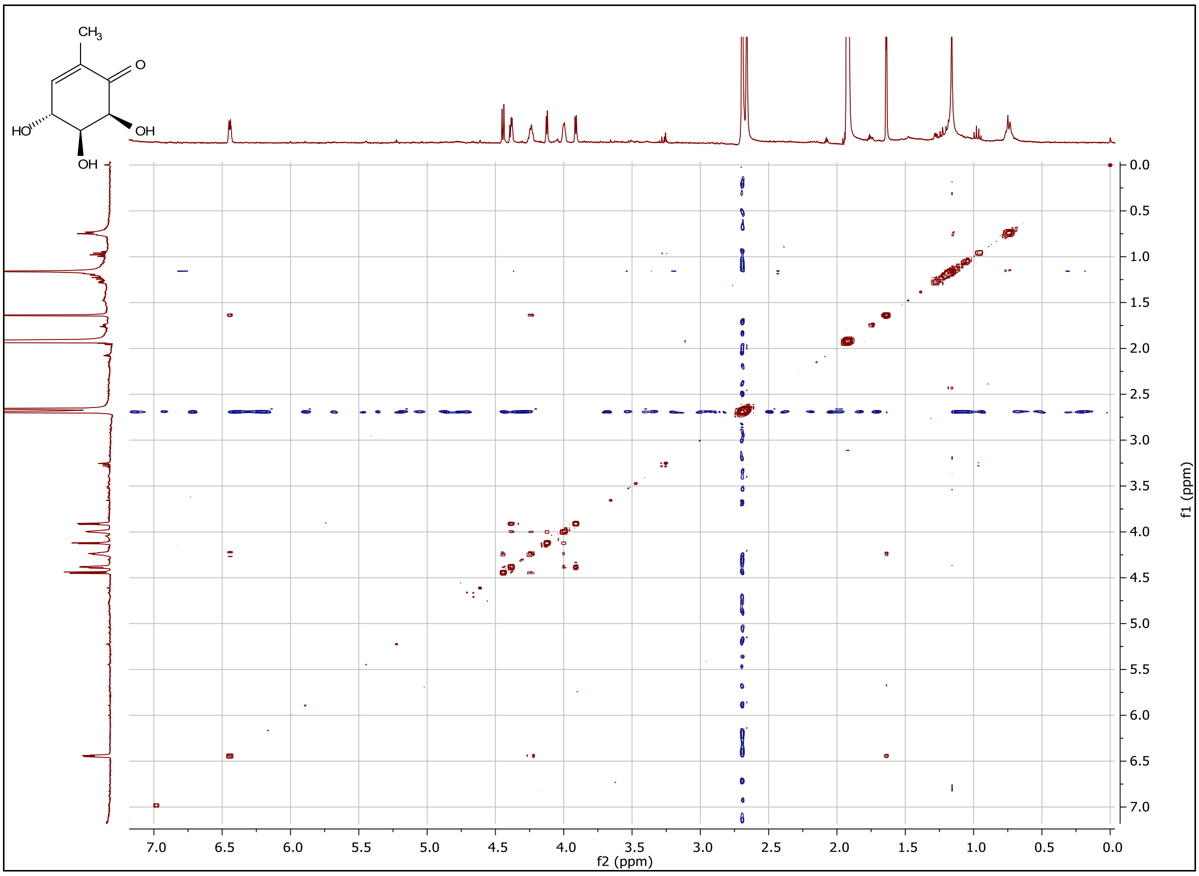

Figure S36. ^{13}C-NMR spectrum of **NNG3**

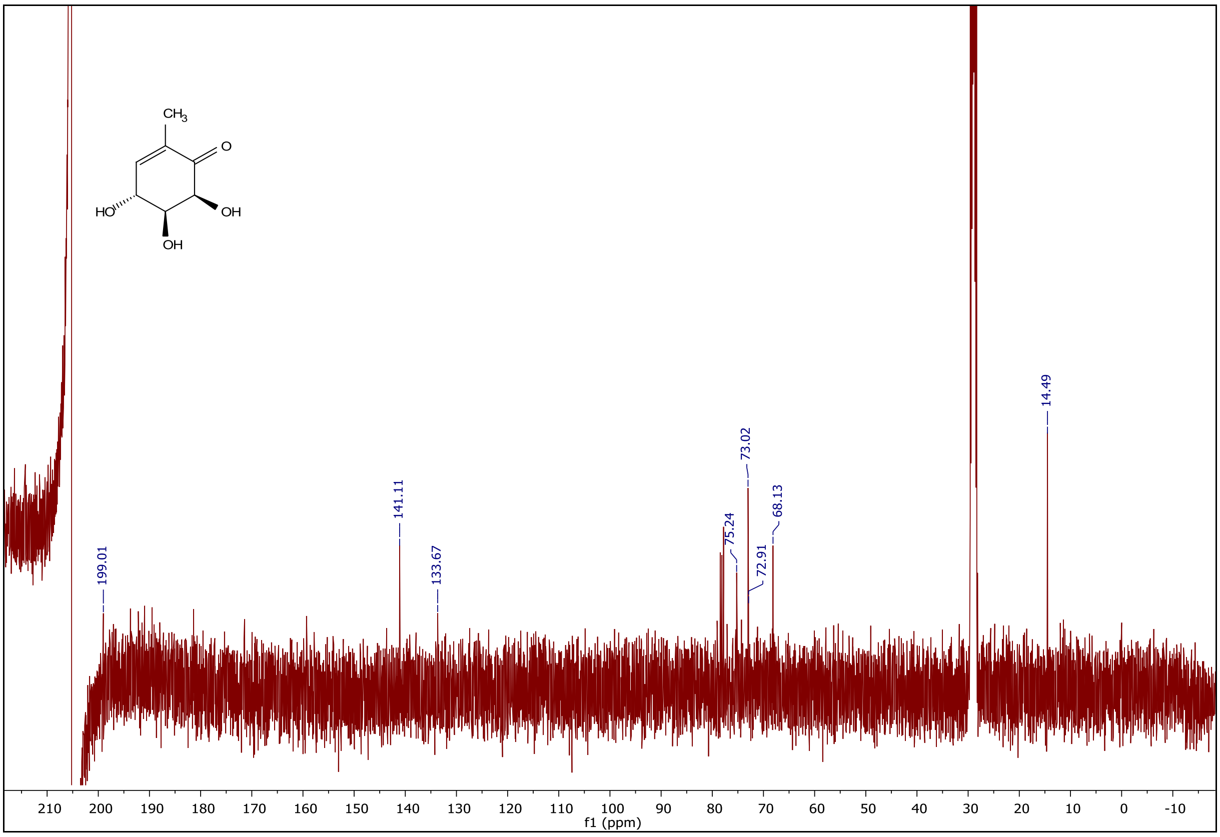

Figure S37. HSQC experiment of **NNG3**

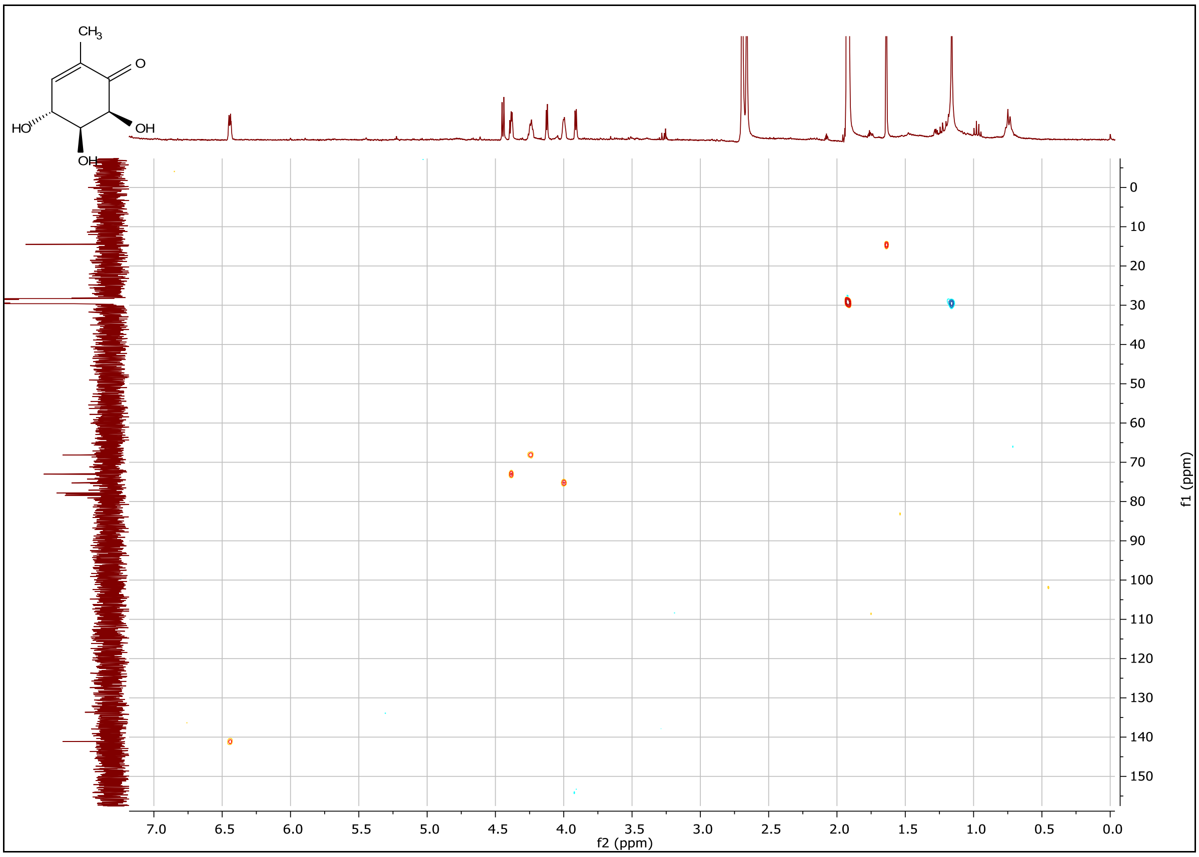

Figure S38. HMBC experiment of **NNG3**

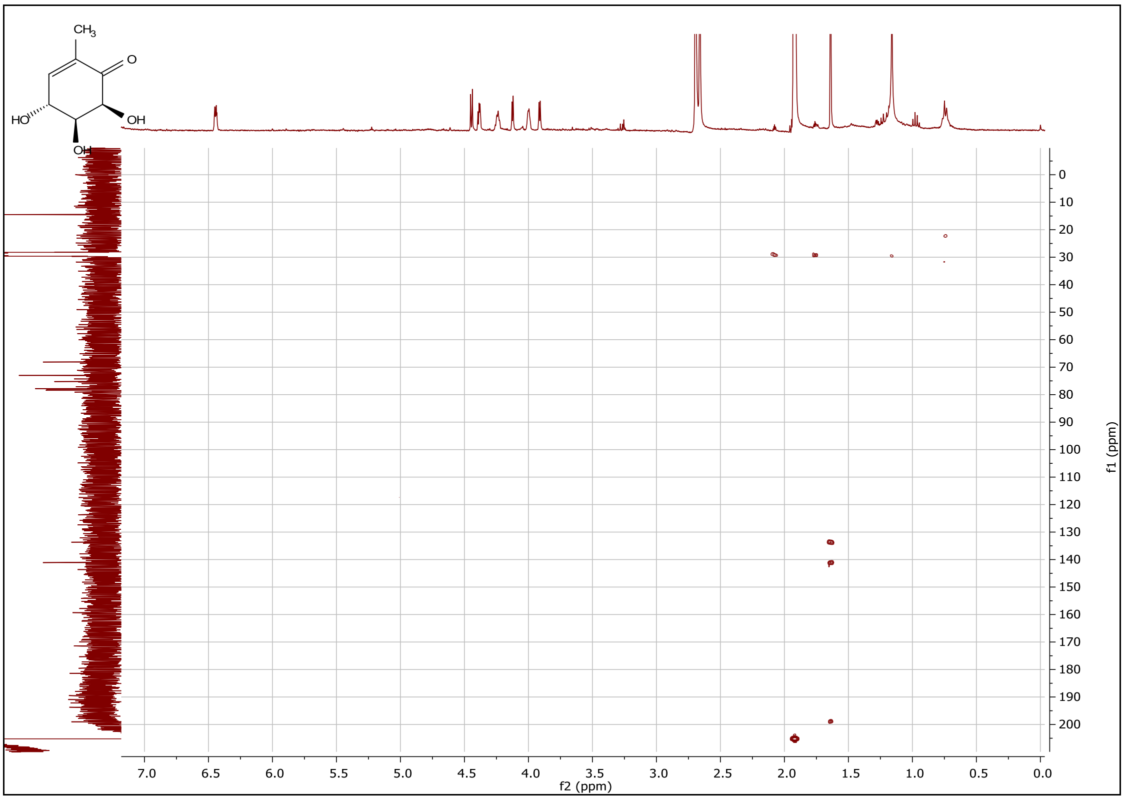

Figure S39. IR spectrum of **NNG3**

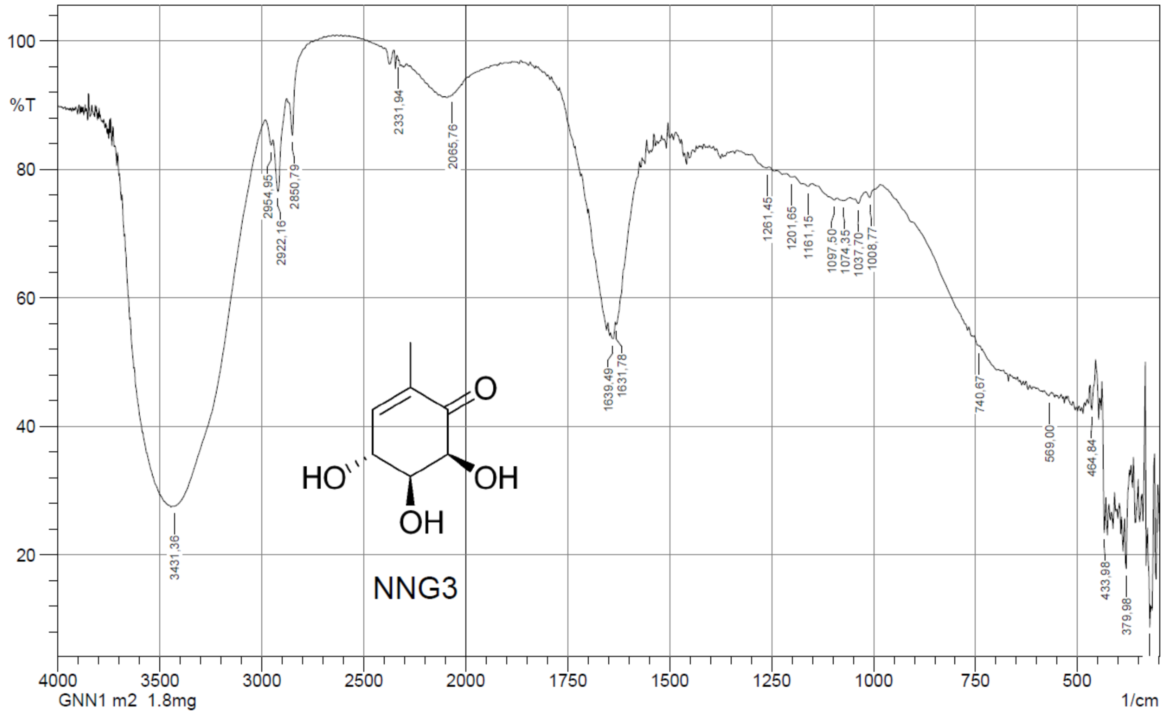

Comment;
GNN1 m2 1.8mg

Date/Time; 29/9/2020 13:01:56
No. of Scans; 100
Resolution; 4 [1/cm]
Apodization; Happ-Genzel
User; usuario

Figure S40: ^{1}H-NMR spectrum of **15**

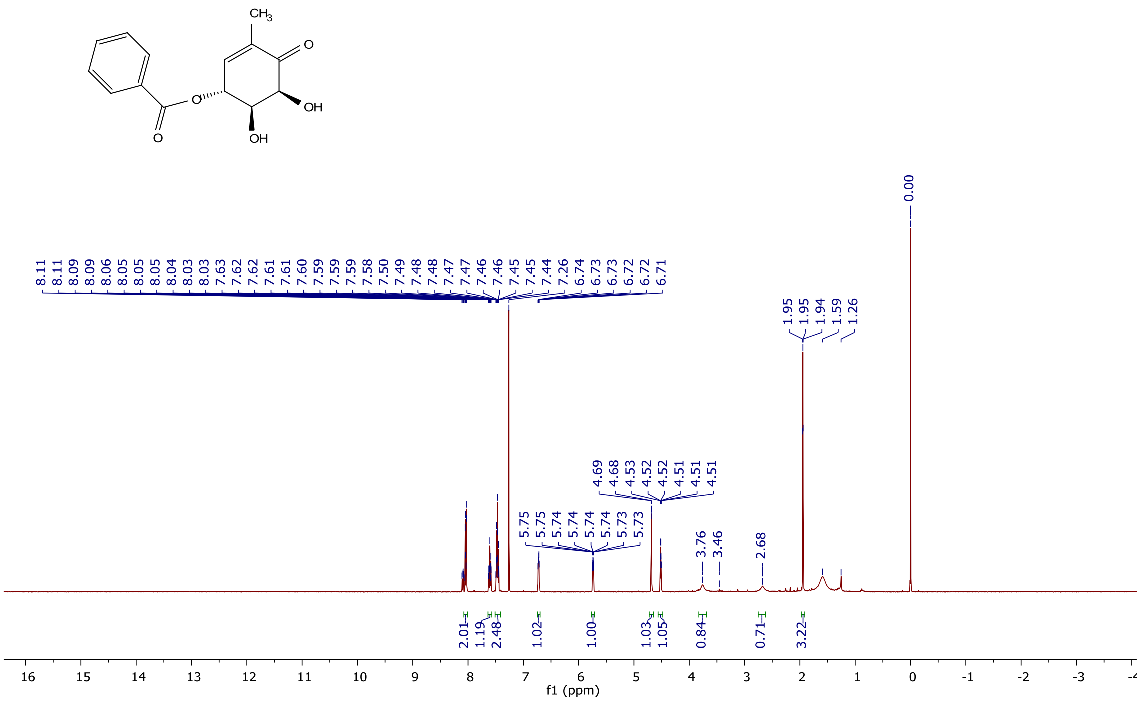

Figure S41: COSY experiment of **15**

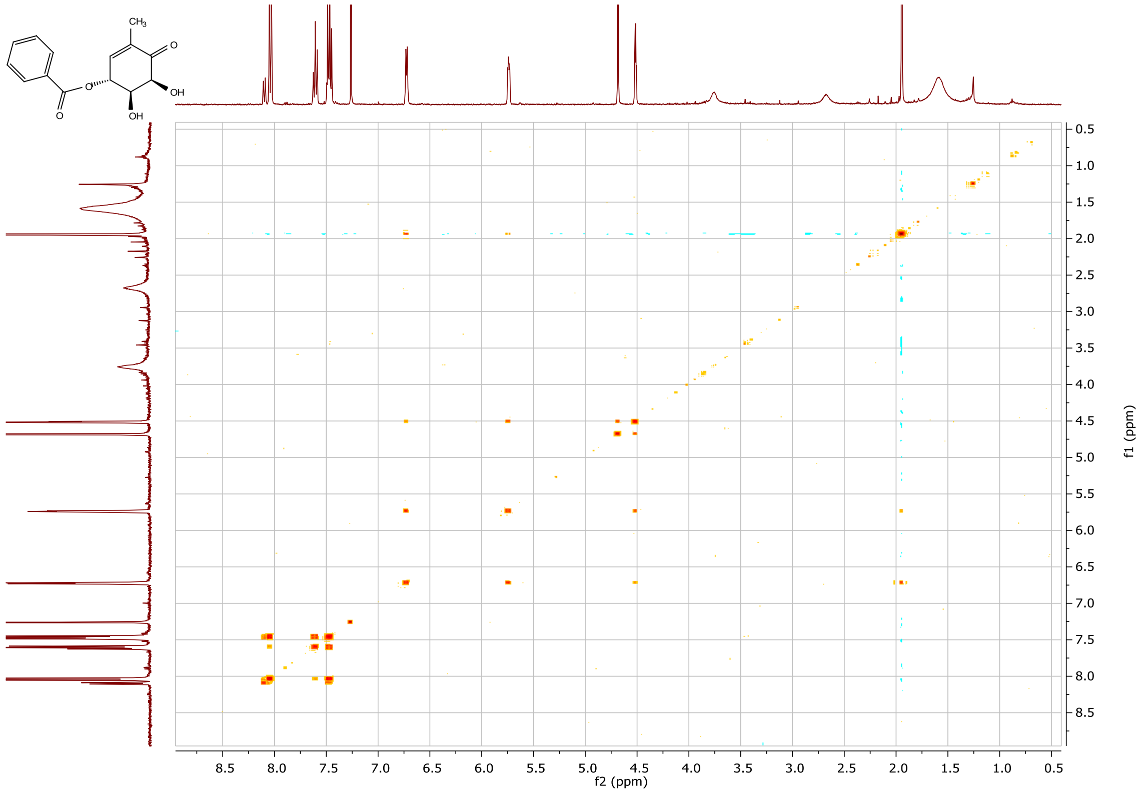

Supplement: Supplementary file 1 [file molecules-26-01423-s001.pdf]
